# Genuine cross-frequency coupling networks in human resting-state electrophysiological recordings

**DOI:** 10.1371/journal.pbio.3000685

**Published:** 2020-05-06

**Authors:** Felix Siebenhühner, Sheng H. Wang, Gabriele Arnulfo, Anna Lampinen, Lino Nobili, J. Matias Palva, Satu Palva

**Affiliations:** 1 Neuroscience Center, HiLIFE-Helsinki Institute of Life Science, University of Helsinki, Finland; 2 Doctoral Program Brain & Mind, University of Helsinki, Finland; 3 BioMag Laboratory, HUS Medical Imaging Center, Helsinki, Finland; 4 Department of Informatics, Bioengineering, Robotics and System engineering, University of Genoa, Genoa, Italy; 5 Child Neuropsychiatry Unit, IRCCS, Istituto G. Gaslini, Department of Neuroscience (DINOGMI), University of Genoa, Italy; 6 Claudio Munari Epilepsy Surgery Centre, Niguarda Hospital, Italy; 7 Department of Neuroscience and Biomedical Engineering, Aalto University, Finland; 8 Centre for Cognitive Neuroimaging, Institute of Neuroscience & Psychology, University of Glasgow, United Kingdom; New York University, UNITED STATES

## Abstract

Phase synchronization of neuronal oscillations in specific frequency bands coordinates anatomically distributed neuronal processing and communication. Typically, oscillations and synchronization take place concurrently in many distinct frequencies, which serve separate computational roles in cognitive functions. While within-frequency phase synchronization has been studied extensively, less is known about the mechanisms that govern neuronal processing distributed across frequencies and brain regions. Such integration of processing between frequencies could be achieved via cross-frequency coupling (CFC), either by phase–amplitude coupling (PAC) or by *n*:*m*-cross–frequency phase synchrony (CFS). So far, studies have mostly focused on local CFC in individual brain regions, whereas the presence and functional organization of CFC between brain areas have remained largely unknown. We posit that interareal CFC may be essential for large-scale coordination of neuronal activity and investigate here whether genuine CFC networks are present in human resting-state (RS) brain activity. To assess the functional organization of CFC networks, we identified brain-wide CFC networks at mesoscale resolution from stereoelectroencephalography (SEEG) and at macroscale resolution from source-reconstructed magnetoencephalography (MEG) data. We developed a novel, to our knowledge, graph-theoretical method to distinguish genuine CFC from spurious CFC that may arise from nonsinusoidal signals ubiquitous in neuronal activity. We show that genuine interareal CFC is present in human RS activity in both SEEG and MEG data. Both CFS and PAC networks coupled theta and alpha oscillations with higher frequencies in large-scale networks connecting anterior and posterior brain regions. CFS and PAC networks had distinct spectral patterns and opposing distribution of low- and high-frequency network hubs, implying that they constitute distinct CFC mechanisms. The strength of CFS networks was also predictive of cognitive performance in a separate neuropsychological assessment. In conclusion, these results provide evidence for interareal CFS and PAC being 2 distinct mechanisms for coupling oscillations across frequencies in large-scale brain networks.

## Abstract 2

Genuine interareal cross-frequency coupling (CFC) can be identified from human resting state activity using magnetoencephalography, stereoelectroencephalography, and novel network approaches. CFC couples slow theta and alpha oscillations to faster oscillations across brain regions.

## Introduction

Human electrophysiological activity is characterized by neuronal oscillations, i.e., rhythmic excitability fluctuations in a wide range of frequencies, at least from 0.01 to over 150 Hz. Synchronization of these oscillations, commonly estimated as phase synchrony (PS), across brain areas coordinates and regulates anatomically distributed neuronal processing [1, 2]. In humans, large-scale oscillatory networks in several frequency bands characterize magnetoencephalography (MEG), electroencephalography (EEG), and stereo-EEG (SEEG) data during resting-state (RS) activity [3–8] and in many cognitive functions [9–13]. Interareal synchronization of alpha (α, 7–14 Hz) and beta (β, 14–30 Hz) oscillations in humans and nonhuman primates, respectively, is thought to regulate top-down or feedback communication [14–19]. In contrast, both β and gamma-band (γ, 30–100 Hz) oscillations and synchronization have been associated with bottom-up sensory processing and representation of object-specific sensory information [15, 20–22], and β oscillations are also associated with sensorimotor processing [23, 24]. Overall, brain-wide oscillation networks in multiple frequencies are proposed to be the core of cognition [10, 11, 25–27]. Also, human brain activity at rest is characterized by resting-state networks (RSNs), first identified with functional magnetic resonance imaging (fMRI) [28, 29]. Oscillatory RSNs observed in electrophysiological measurements are organized in a partially similar fashion as the RSNs observed with fMRI [3, 30] as well as into a modular structure at the whole-brain connectome level [31]. It has been proposed that RSNs form the basis of task-state large-scale networks [8, 32].

**Fig 1:**
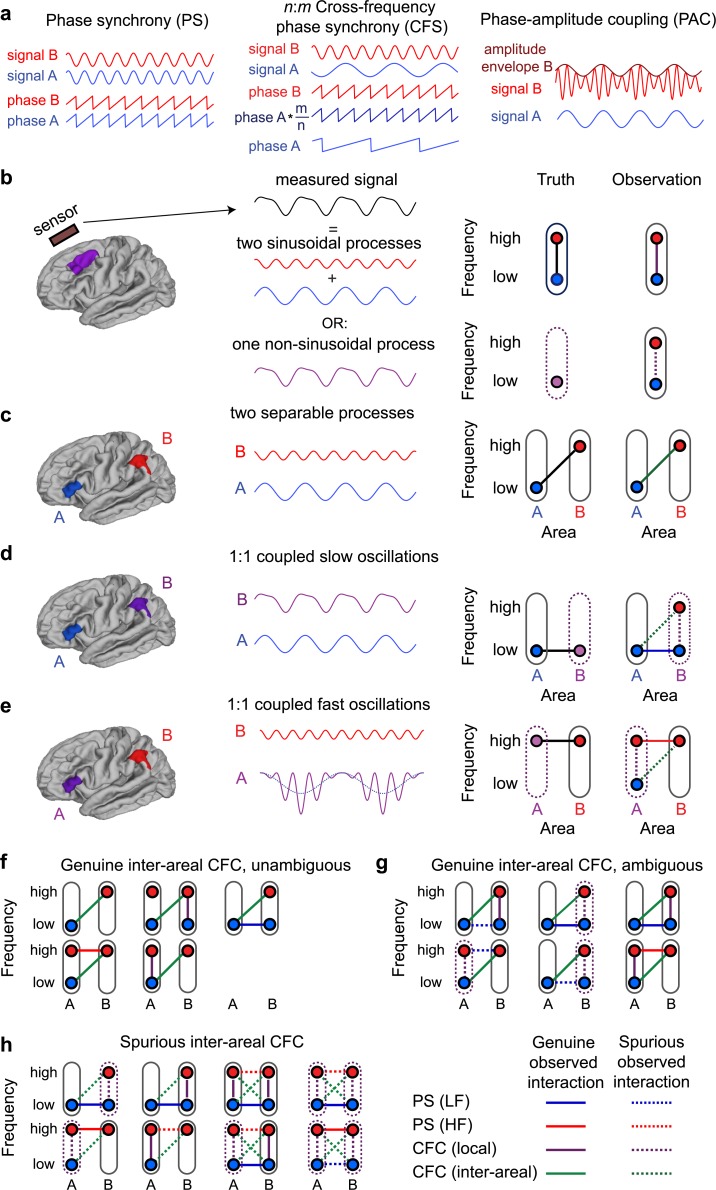
Schematics of identifying genuine and spurious interareal CFC. (a) Schematic illustration of PS, LF:HF (*n*:*m*) CFS, and LF:HF PAC. In PS, 2 spatially distant processes oscillating at the same frequency exhibit a (statistically) constant phase relationship. In CFS, a constant *n*:*m*-phase relationship exists between 2 processes at frequencies LF and HF, so that LF:HF = *n*:*m*. In PAC, the amplitude of the HF signal is correlated with the phase of the LF signal. These processes can either take place in the same region (local CFC) or between 2 regions (interareal CFC). (b) Observations of local CFC can be either genuine or spurious. A measured signal from a single sensor or electrode can either be the sum of 2 (statistically) sinusoidal processes oscillating at distinct frequencies or a single nonsinusoidal process, and these possibilities are difficult to dissociate from the single signal. Local CFC can be observed in both cases because of filter artifacts produced by nonsinusoidal signals. (c) Genuine interareal CFC between 2 spatially distant sinusoidal processes A and B. (d) An example of spurious observation of interareal CFC. Process A is sinusoidal, but B is nonsinusoidal and causes spurious local CFC to be observed at location B, as shown in (b). If A and B are connected by LF PS, spurious interareal CFC will also be observed between A and B. This spurious observed interareal CFC forms a “triangle motif” with PS and the spurious CFC couplings. (e) Example of spurious observation of interareal CFC in which process B is sinusoidal, but A has a nonzero mean, and spurious local CFC will be observed at location A. Again, if A and B are connected by HF PS, spurious interareal CFC will also be observed between A and B, again forming a triangle motif. (f) Constellations of observations that unambiguously indicate genuine CFC between regions A and B. In none of the cases is there a triangle motif of PS and local CFC. (g) Constellations of observations with ambiguous finding of interareal CFC between regions A and B. Although here, interareal CFC is genuine, it is part of a triangle motif formed with PS and (true or spurious) local CFC. Such constellations cannot be distinguished by our graph-theory–based method from spurious interareal CFC. (h) Constellations with spurious interareal CFC. These include the 2 constellations from (d) and (e) in the left column and other possible constellations, including those in which there is spurious local CFC at both locations (right column). In all cases, the spurious interareal CFC is part of a triangle motif. CFC, cross-frequency coupling; CFS, cross-frequency PS; HF, high frequency; LF, low frequency; PAC, phase–amplitude coupling; PS, phase synchrony.

The interplay between oscillations at distinct frequencies is thought to be regulated via 2 forms of cross-frequency coupling (CFC): phase–amplitude coupling (PAC) [9, 33–37] and cross-frequency phase synchrony (CFS) [9, 38–41], also known as *n*:*m*-PS [38]. PAC indicates the correlation between the amplitude envelope of a faster oscillation and the phase of a slower oscillation, whereas CFS is a form of phase synchronization defined by a nonrandom phase difference between oscillations having an integer *n*:*m* frequency ratio (Fig 1A). During task performance, PAC has been suggested to reflect the regulation of sensory information processing in β- and γ-frequencies by excitability fluctuations imposed by θ and α oscillations [9, 10, 34, 36, 37, 42]. A large number of studies have identified local PAC, i.e., PAC observed between different frequency bands of the same signal, between the phases of slower oscillations in delta- (δ, 1–3 Hz), theta- (θ, 3–7 Hz), and α-frequency bands and the amplitude of γ oscillations in local field potentials (LFPs) in rats [43–47] and in human intracranial EEG [48–51] and MEG data [52–56]. Such local PAC has cortex-wide spatial modes akin to RSNs [53]. Unlike PAC, CFS enables temporally precise coordination of neuronal processing by establishing systematic spike-timing relationships among possibly functionally distinct oscillatory assemblies and hence has been suggested to serve functional integration and coordination across within-frequency synchronized large-scale networks [39, 41, 57]. Local CFS has been observed in human MEG and EEG data during rest [39, 58] and during attentional and working memory (WM) tasks [39, 40, 59] as well as in LFPs in the rat hippocampus [45]. However, observations of CFS have remained scarcer than those of PAC, and it has remained unclear whether CFS and PAC are even distinct phenomena or simply different reflections of a single CFC phenomenon. Some studies have also identified interareal PAC or CFS between a few preselected regions of interest between MEG/EEG sensors [39, 40] or between brain regions in source-reconstructed MEG/EEG data [41, 53, 56] or intracranial data [60]. However, only a few studies [39, 41] have identified CFC in the cortex-wide networks that form the basis of cognitive functions. Therefore, it has remained largely unknown whether CFC can couple oscillations across frequencies in large-scale brain networks and, if so, what is the functional organization of these networks, their similarities to PS networks, and their relevance for cognitive performance and abilities. Furthermore, recent research has suggested that estimates of CFC may be inflated by false-positive couplings arising from nonsinusoidal and nonzero mean signals. False positives are caused by the artificial higher-frequency components produced when nonsinusoidal signals are filtered into narrow bands [61–68], as well as by artificial lower-frequency components arising from filtering of non-zero–mean waveforms [69]. Because nonsinusoidal and non-zero–mean waveforms are ubiquitous in electrophysiological signals, their filter artifacts constitute a significant confounder to CFS and PAC estimation and question the validity of prior CFC observations. Because CFC is thought to be central in the coordination of processing across frequencies, it is crucial to establish whether genuine neuronal CFC can be observed in neuronal activity in the first place.

The fundamental assumption in CFC is that it indicates an interaction between 2 distinct neuronal processes in 2 frequency bands. Conversely, the notion of artificial CFC arising from nonsinusoidal signal properties relies on the assumption that a neuronal process exclusively in a single frequency band generates the observed signal. Approaches based on waveform analysis [63, 68, 70] and appropriate control analyses [69] have been proposed to reduce the artifactual connections. Nevertheless, filter-artifact–caused spurious CFC, in particular CFS, is difficult to dissociate from genuine CFC by inspection of the waveform shape of any single signal in isolation. Local CFC estimates are thus prone to ambiguous results. However, CFC is necessarily genuine when there is evidence for 2 distinct coupled processes. Building on this notion, we advance here a conservative test to identify genuine CFC, i.e., one that minimizes false positives, based on connection-by-connection testing of whether CFC can unambiguously be attributed to 2 separable processes.

In this study, our objectives were (i) to investigate whether genuine interareal CFC between brain regions characterizes meso- and macroscale RS activity in human SEEG and source-reconstructed MEG, respectively; (ii) to reveal the functional organization of these networks; (iii) to test whether the 2 predominant forms of CFC, PAC and CFS, were phenomenologically similar; and (iv) to investigate whether the strength of RS CFC is predictive of cognitive performance. We estimated whole-brain connectomes of CFS and PAC and identified anatomical and topological structures therein. With SEEG data, we further addressed the putative distinct roles of generators in deep and superficial layers. We found that genuine interareal CFS and PAC indeed characterize human RS activity in both SEEG and MEG after pruning of connections that could be artifact-related false positives. CFS and PAC networks were characterized by directional coupling between the prefrontal, medial, visual, and somatomotor (SM) cortices, but crucially, with distinct spectral profiles and opposing directionalities. The strength of large-scale CFS RSNs was also predictive of cognitive performance in neuropsychological assessments. These results reveal the organization of genuine CFC in human RS brain activity and provide evidence for CFS and PAC being functionally distinct mechanisms in the coupling of neuronal oscillations across frequencies.

## Results

### A graph-theory–based method for identifying genuine neuronal interareal CFS and PAC

Our first objective was to assess the presence and large-scale organization of genuine CFC in human RS brain activity at mesoscale resolution with SEEG and at macroscale resolution with source-reconstructed MEG data. In order to systematically address the possibility that observations of CFC might be spuriously caused by filtering artifacts stemming from nonsinusoidal or non-zero–mean waveforms, we advance here a new, to our knowledge, method to control for spurious connections. Our method is based on the core assumption that any genuine CFC interaction takes place between 2 distinct processes, whereas spurious CFC is a property of a single process with signal components distributed to distinct frequency bands because of filter artifacts from nonsinusoidal or non-zero–mean signal properties. Thus, we set out to test whether observations of interareal CFC reflect origins in 2 separable processes or within 1 process. In our graph-theoretical, network-motif–based approach, we assess for each observation of interareal CFC between areas A and B whether there is also observed interareal within-frequency PS and local CFC that together may lead to a spurious observation of interareal CFC. If this is the case, the observed interareal CFC possibly does not connect distinct oscillatory processes and may hence be spurious, whereas the absence of either PS or local CFC indicates that the observed interareal CFC cannot be attributable to a single source and is thus genuine.

Fig 1 shows basic schemata for PS, CFS, and PAC (Fig 1A) and our approach for dissociating the genuine from putatively spurious observations. Spurious observations of local CFC occur when a nonsinusoidal signal is filtered, which creates coupled signals at distinct frequencies that cannot be easily distinguished from genuine observations of local CFC (Fig 1B). On the other hand, interareal CFC, connecting locations giving rise to separable signals, can be proven to be genuine if it can be shown that the signals unambiguously originate from separable neuronal processes (Fig 1C). Spurious interareal CFC may arise only when spurious local CFC is observed at one or both locations that are also interareally coupled via 1:1 PS either at low frequency (LF) or high frequency (HF) so that a “triangle motif” with the observed (spurious) interareal CFC is formed (Fig 1D and 1E). We therefore developed a graph-theory–based method to identify all CFC–PS network triangle motifs that might contain spurious interareal CFC (Fig 1F–1H). This approach only identifies those interareal CFC observations as genuine that are not part of a full triangle motif (Fig 1F), whereas all others are discarded. Because this may include also genuine connections (Fig 1G) among the spurious ones (Fig 1H), our approach is conservative and provides a lower bound for the number of genuine connections. Notably, also cases wherein there is nonsinusoidal activity at both locations are excluded (Fig 1H, right half).

### Simulation of CFC and spurious interactions with the Kuramoto model

We thus posit that genuine CFC may be unambiguously observed between sources that are anatomically separable because that enables the separation of the LF and HF processes. The central statistical consideration in this is that observations of significant interareal CFC may also spuriously arise from the combination of adequately strong interareal 1:1 phase synchronization and local CFC, either genuine or artificial. Such spurious interareal CFC should, however, always be weaker than the local CFC because it arises only indirectly from statistical regularities.

**Fig 2:**
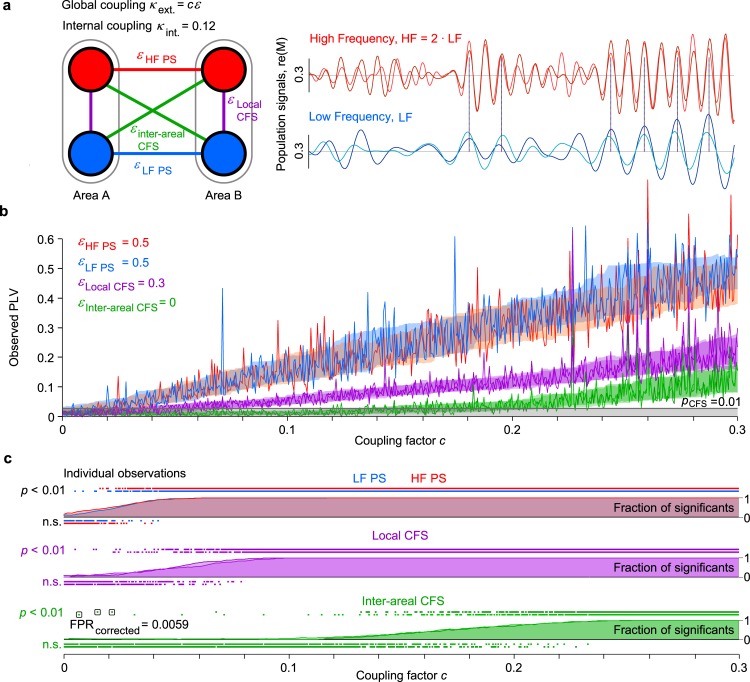
Generative modeling of joint 1:1 and 1:2 cross-frequency phase coupling. (a) Two areas (A and B), each containing 2 populations of Kuramoto oscillators (*N* = 500) at LFs and HFs. The populations exhibit intermediate and intermittent internal synchronization (see time series) and are mutually coupled by population-signal–based 1:1 PS or 1:2 CFS phase coupling (see *ε*). (b) Increasing the 1:1 and local CFS coupling between populations led to strengthening of the corresponding phase correlations (PLV, red and blue lines for PS and purple for local CFS) and, in the regime of strong coupling, also to the emergence of spurious interareal CFS (green lines). Each data point indicates the observed phase correlation (PLV) in a single simulation with 100,000 iterations (5,000 cycles of the fast oscillation) with random initial parameters in a series of 512 simulations for coupling factors from 0 to 0.3. Shaded areas indicate the 16th to 84th PLV percentiles across simulations. The gray line and shaded area indicate the PLV threshold for nominally significant CFS at *p* < 0.01. (c) Phase correlation statistics: small squares indicate significant (*p* < 0.01) or n.s. phase correlation observations in individual simulations in the example of series of panel (b). Lines and the shaded areas indicate the fraction of significant observations as a function of the shared coupling factor *c*. Black frames indicate the observations of interareal CFS that were not associated with significant local CFS and 1:1 PS and would thus remain as false positives after the correction proposed in this study. CFC, cross-frequency coupling; CFS, cross-frequency PS; FPR, false positive rate; HF, high frequency; LF, low frequency; n.s., not significant; PLV, phase-locking value; PS, phase synchrony.

To test this rigorously, we developed a generative 4-population Kuramoto model [71] for investigating the joint effects of within- and cross-frequency phase coupling (see Methods, Modeling). The model comprised 2 “areas” that each contained 2 populations of weakly coupled oscillators: one at LF and another at HF, so that *f*_HF_ = 2 × *f*_LF_, i.e., with the *n*:*m* ratio (hereafter defined as LF:HF ratio) of 1:2 (Fig 2A). The populations were coupled with coupling strengths *ε* with each other via 1:1 PS, local CFS, and interareal CFS, and these couplings varied with a shared coupling factor *c*. The model produced salient 1:1 and 1:2 phase coupling at large coupling values (all *ε* = 0.5, *c* = 0.3) with biologically plausible intermittent synchronization dynamics (Fig 2A, right).

To investigate the emergence of spurious interareal CFS, we simulated interareal PS and local 1:2 CFS with zero genuine interareal CFS. Hence, here all observations of interareal CFS were spurious and driven by the indirect joint effect of local CFS and interareal PS (Fig 2B). This analysis showed that as 1:1 PS and local 1:2 CFS increased (see Fig 2B, *c* > 0.02), interareal 1:2 CFS was indeed also observed in increasing strength. The crucial test for our method was to then inspect the significant (nominal *p* < 0.01) observations of interareal CFS. For each such observation, we tested whether it would be excluded by a simultaneous observation of significant local CFS and significant interareal PS. We found that this was, by and large, the case (Fig 2C). Because interareal CFS arose through indirect effects of local CFS and interareal PS that reached significance at much lower coupling values, essentially all spurious interareal CFS observations were correctly rejected (example false positives encircled in Fig 2C, bottom panel). The nominal false-positive rate (FPR) was 0.006 ± 0.002 (mean ± SD) across the simulations, and these false positives were attributable to the very-low–coupling regime in which both local and interareal CFS were at chance level. Hence, for couplings above chance level, the method proposed here is effective in pruning putative spurious observations of interareal CFS.

### Interareal CFC in single-subject SEEG and MEG analyses

To estimate the presence of genuine interareal CFC and to map the functional organization of CFC networks, we used eyes-closed RS SEEG data (10 minutes, 59 subjects) from epileptic patients and eyes-open RS MEG data (10 minutes, 19 subjects) from healthy controls (for the analysis workflow, see S1 Fig, S2 Fig). SEEG data analysis was performed at the level of individually localized electrode contacts, from which we excluded those located in the epileptic zone or exhibiting large artifacts (see Methods, SEEG data acquisition and SEEG data preprocessing and filtering). For the MEG subjects, MRI scans were also obtained and used for generating individual cortical source models for source reconstruction (see Methods, MEG and MRI data acquisition). We obtained for each subject a cortical parcellation (see Methods, Cortical parcellation) of 200 cortical parcels by iteratively splitting [72] the largest parcels in the Destrieux atlas [73]. For both SEEG and MEG, the broadband time series were filtered into narrow-frequency bands from 1 to 315 Hz. For MEG data, these were then inverse modeled and collapsed to cortical-parcel time series (Methods, Source model and colocalization, MEG data preprocessing and filtering, and MEG source reconstruction: Inverse transform and collapsing of source signals to parcel time series). We excluded from further analyses parcels and parcel–parcel connections for which the source-reconstruction and connection-detection accuracy were poor, respectively (see Methods, Removal of low-fidelity parcels and connections from MEG connectivity analysis). From these data, we estimated interareal CFC between all remaining SEEG electrode contacts and MEG parcels. CFC was estimated for LF time series between 1–95 Hz and HF time series between 2–315 Hz at LF:HF ratios of 1:2–1:7. For the removal of the spurious connections as described above, we also estimated interareal 1:1 PS and amplitude envelope correlations (ACs) between pairs of electrode contacts or parcels (see Methods, Analysis of interareal phase synchronization, Analysis of local and interareal CFC: PAC and CFS, and Analysis of amplitude–amplitude coupling).

**Fig 3:**
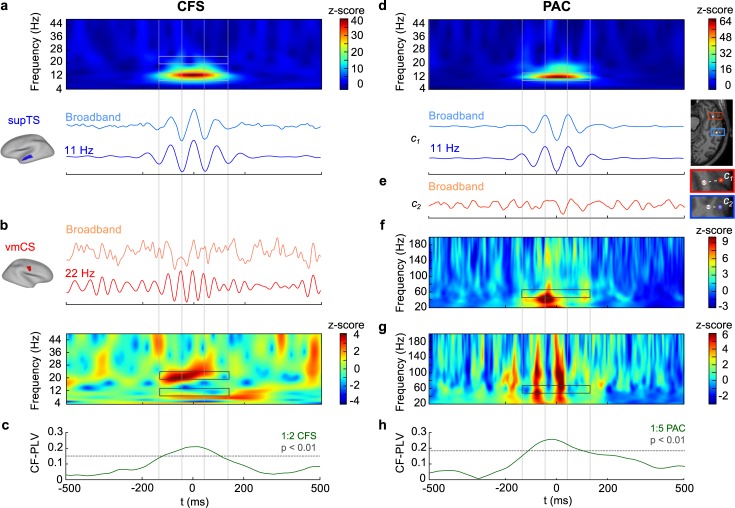
Genuine interareal CFS and PAC at the single-subject level. (a) Averaged broadband time series and α**-**band (11 Hz)–filtered LF time series time-locked to the α peaks in the left supTS for a representative MEG subject. TFR of the broadband average reveals α oscillations (highlighted by lower gray box) but an absence of β oscillations (in area of upper gray box) and thus the absence of both nonsinusoidal filter artifacts and genuine local 1:2 CFS. (b) Averaged broadband-filtered and β-band (22 Hz)–filtered HF time series for right vmCS time-locked to the α LF peaks identified in supTS. TFR of the broadband average reveals β oscillations but an absence of α oscillations. Thus, vmCS shows no filter artifacts, local CFS, or interareal α or β CFS (highlighted by gray boxes). (c) PLV time series (green line) for 1:2 α:β CFS for the LF and HF time series averaged over α-peak time-locked segments. Dotted gray line shows the PLV value above which CFS is significant at *p* < 0.01. (d) Averaged broadband-filtered and α**-**band (11 Hz)–filtered LF time series time-locked to the α troughs in an electrode contact *c*_1_ located in the mTG in a representative SEEG subject. The electrode location is marked by the red circle on the brain, and the closest white matter contact (used as reference) by the white circle next to it. TFR of the averaged broadband time series reveals α oscillations (highlighted by gray box) and an absence of higher-frequency components, suggesting the absence of systematic nonsinusoidal filter artifacts that would show up as local CFS here. (e) Averaged broadband time series in an electrode contact c_2_ in iTS that is time-locked to the α troughs in contact c_1_ reveals no clear α oscillations, showing an absence of α PS between *c*_1_ and *c*_2_. The location of electrode contact c_2_ is marked by the blue circle on the brain, and the closest white matter contact (reference) by the white circle next to it. (f) TFR of oscillation amplitudes in *c*_1_ time-locked to α peaks shows little modulation of γ amplitudes by α phase at frequencies above 40 Hz. (g) TFR of oscillation amplitudes in *c*_2_ show comodulation of γ amplitudes in *c*_2_ and α cycles (i.e., α phase) in *c*_1_. The frequency region at around 55 Hz, at which the HF of the 1:5 PAC should be seen, is marked with gray boxes. (h) PLV time series (green line) for 1:5 PAC between LF time series in *c*_1_ and LF-filtered amplitude envelope of 55 Hz NB in *c*_2_. Dotted gray line shows the PLV value above which PAC is significant at *p* < 0.01. Plot data for a–h are available online at https://datadryad.org/stash/dataset/doi:10.5061/dryad.0k86k80. CFS, cross-frequency PS; HF, high frequency; iTS, inferior temporal sulcus; LF, low frequency; MEG, magnetoencephalography; mTG, middle temporal gyrus; NB, narrowband; PAC, phase–amplitude coupling; PLV, phase-locking value; PS, phase synchrony; SEEG, stereoelectroencephalography; supTS, superior temporal sulcus; TFR, time frequency representation; vmCS, ventromedial central sulcus.

To first acquire a proof of concept for genuine CFC at the single-subject level, we identified single-subject data sets with strong CFC. We selected an MEG participant with strong interareal CFS between alpha (α) and beta (β) oscillations with a ratio of 1:2 (α:β CFS) and an SEEG participant with strong interareal PAC between α and gamma (γ) oscillations (α:γ PAC) with a ratio of 1:5. We focused on observations of interareal CFC between areas that were not connected by interareal PS and/or local CF in the triangle motif so that CFC between them was genuine from the perspective of our approach (see Methods, Removal of potentially spurious CFC connections). To then measure CFS in an independent manner that allows the dissection of filter artifacts from genuine coupling, we averaged unfiltered data segments time-locked to the peaks of narrowband (NB)-filtered α oscillations in the temporal sulcus (TS) (see Methods, Single-subject analysis of CFC). Time-frequency (TF) analysis of the average signal revealed a peak only in the α-band, showing that neither genuine nor spurious local CFS was observable therein (Fig 3A). We next used the same α-oscillation peak latencies to average broadband signals from another source, the central sulcus (CS; Fig 3B). TF analysis of the peak-averaged broadband data in the CS revealed an oscillation in the β-band, matching averaged β-band–filtered data. However, no components in the α-band in CS were found in TF analysis, which confirmed the absence of both local CFS therein and interareal α PS between TS and CS. The observation of α-peak locking of β oscillations between these 2 regions thus unambiguously indicated genuine interareal CFS coupling. As a confirmatory analysis, we estimated time-resolved α:β CFS between these 2 locations and found α:β CFS to be significant at *p* < 0.01 for a duration of approximately 300 ms around the α peak in the TS (Fig 3C).

We then adopted this approach to assess local and interareal PAC. We first detected α troughs from SEEG data and averaged data segments time-locked to these troughs in the electrode contact *c*_1_ located in the middle temporal gyrus (mTG). Both the averaged broadband time series itself and its TF analysis showed only α oscillations (Fig 3D). The broadband signal in an electrode contact *c*_2_ in the inferior temporal sulcus (iTS), time-averaged to the α troughs identified in the first contact, revealed no salient α oscillations, showing that these 2 contacts were not coupled by α PS (Fig 3E). Amplitude TF analysis, i.e., averaging of the NB amplitude envelopes around the α peaks in *c*_1_, revealed no evidence of local PAC in *c*_1_ (Fig 3F) save for a peak at around 40 Hz during one α cycle. However, in *c*_2_, γ amplitude was comodulated by multiple *c*_1_ α cycles over a wide range of frequencies, including 55 Hz (which was the initial finding), indicating the presence of true interareal PAC (Fig 3G). As a confirmatory analysis, we then evaluated time-resolved 1:5 α:γ PAC between LF 11 Hz at *c*_1_ and HF 55 Hz at *c*_2_ (frequencies indicated by the gray boxes in Fig 3D and Fig 3F and 3G, respectively) and found that PAC was significant for nearly 3 α cycles at *p* < 0.01 around the central α trough (Fig 3H).

### Connectomes of interareal CFS and PAC in SEEG and MEG data

**Fig 4:**
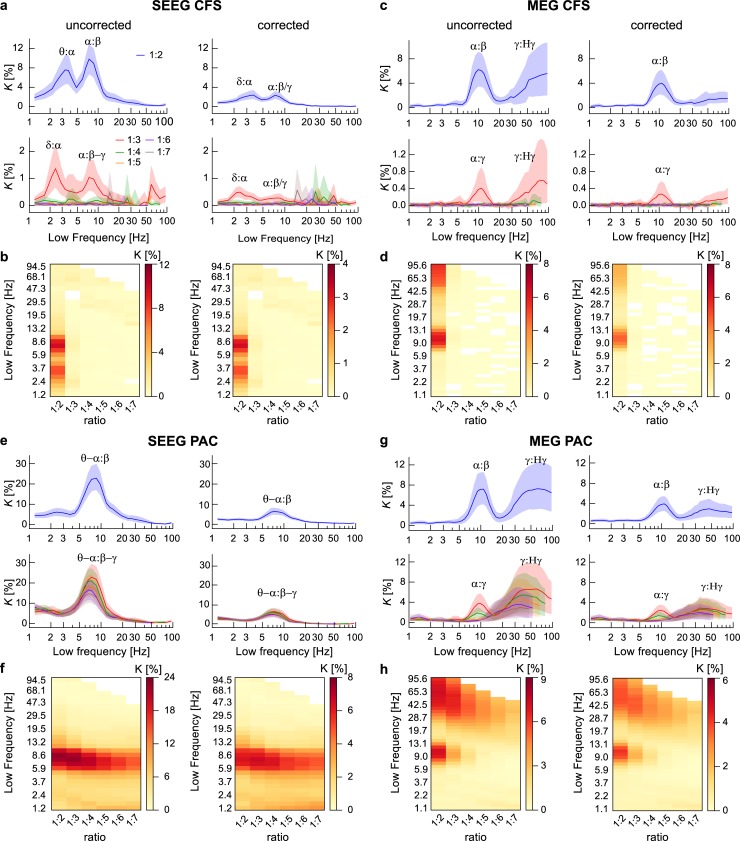
Genuine observations of interareal CFS and PAC. (a) Connection density (*K*), i.e., the fraction of significant connections over all possible connections, of interareal CFS in SEEG at the group level before (left) and after removing possible spurious connections (right) for LF:HF ratio 1:2 (top row) and ratios 1:3–1:7 (bottom row). The *x* axis shows the LF. *K* values are plotted with 95% confidence limits obtained from surrogate data. (b) The same data as in (a), but presented in a matrix in which each frequency-ratio combination is a matrix element. *K* is again presented before (left) and after removal of possibly spurious connections (right). (c–d) Interareal CFS in MEG before and after removing possibly spurious connections. Robust α:β CFS at ratio of 1:2 and α:γ CFS at ratios 1:3 characterize SEEG and MEG data before removing spurious connections. Although *K* is reduced by removing the putatively spurious connections, α:β at 1:2 ratio and α:γ CFS at 1:3 ratio remain significantly above zero. (e–f) Interareal PAC in SEEG data and (g–h) in MEG data before and after removing spurious connections as in (a)–(d). SEEG is characterized by robust PAC of θ–α oscillations to HFs in α–γ bands at ratios 1:2–1:7, indicating that α–γ band amplitudes are modulated by phases of θ–α oscillations. In MEG, PAC is observed between α phase and β–γ band amplitudes at ratios 1:2–1:4, as well as between γ and Hγ oscillations at all ratios. The connection densities are reduced by removing putatively spurious connections but remain significantly above the zero. Plot data and underlying connectome data are available online at https://datadryad.org/stash/dataset/doi:10.5061/dryad.0k86k80. CFS, cross-frequency PS; HF, high frequency; LF, low frequency; MEG, magnetoencephalography; PAC, phase–amplitude coupling; PS, phase synchrony; SEEG, stereoelectroencephalography.

With both theoretical support for our method and experimental proof of concept for CFS and PAC, we mapped the CFC connectomes (i.e., CFS and PAC between all parcels/channels) for each subject, for all LFs between 1–95 Hz and for all LF:HF frequency ratios 1:2–1:7, in both SEEG (*N* = 59) and MEG (*N* = 27) data. In order to first quantify the prevalence of significant CFS and PAC connections in the SEEG and MEG data, we compared the CFS and PAC connectomes against individually generated surrogate data and identified statistically significant (*p* < 0.01) connections separately for each subject (see Methods, Group-level statistics). We denoted the proportion of significant CFS and PAC connections from all possible connections as the connection density, *K*. To represent these data at the group level, we averaged the individual *K* values and plotted group-level connection density spectra (*K*) as a function of LF separately for each LF:HF ratio (Fig 4; shaded areas indicate 95% confidence limits of the mean estimated by bootstrap resampling). *K* spectra thus summarize the group-mean extent of significant CFC in individual cortical networks.

In both SEEG (Fig 4A and 4B) and MEG (Fig 4C and 4D), the connection density spectra revealed an LF α peak at ratios 1:2 and 1:3, which indicates significant CFS coupling between α with β and γ oscillations, i.e., α:β and α:γ CFS. In SEEG, the frequency range of this peak was approximately 6–12 Hz and in MEG, 7–15 Hz. This elevated amount of significant interareal α:β and α:γ CFS was also reflected as a peak in the corresponding graph strength (GS), which shows that these CFS connections were also stronger in terms of the phase-locking strength (S3 Fig). We also observed similar α-band peaks in *K* spectra of interareal within-frequency PS (S4 Fig) and in estimates of local CFS (S5 Fig). In addition to α-oscillation–based CFS, we found in SEEG LF peaks in the range 2–5 Hz, covering parts of both delta (δ) and theta (θ) bands (Fig 4A and 4B). These δ–θ oscillations were synchronized at ratios 1:2 and 1:3 with θ–α oscillations, although no δ–θ peak was found in the within-frequency PS analysis (S3 Fig). In MEG, on the other hand, CFS was significant also among γ and high-γ bands at ratios 1:2 and 1:3, although the wide confidence limits indicated large interindividual variability (Fig 4C and 4D).

We next assessed interareal PAC with the same approach as above and found significant PAC in both SEEG (Fig 4E and 4F) and MEG (Fig 4G and 4H). As the most salient peak in the connection density spectra, we found that PAC coupled the phase of θ–α band oscillations (5–12 Hz) with the amplitude envelopes of oscillations at higher frequencies. PAC in SEEG was robust throughout the studied range of LF:HF ratios and coupled θ–α oscillation phases with the amplitude of neuronal activity up to the Hγ band. In MEG, α-oscillation phases (7–12 Hz) were coupled with β and γ amplitudes at ratios 1:2–1:4. In MEG, PAC also coupled γ and Hγ band oscillations, similarly to CFS. On the other hand, the δ–θ band oscillations that were coupled via CFS with α oscillations in SEEG were not observed to exhibit PAC with these or higher frequencies, indicating that the observed δ–θ:α coupling in SEEG was specific to CFS. Overall, these data suggest that robust interareal CFC, both CFS and PAC, of θ and α oscillations with oscillations in β and γ frequencies is characteristic to human RS brain activity.

### Genuine interareal CFS and PAC in RS brain activity

We addressed then whether the findings of CFC were attributable to filtering artifacts or reflected genuine neuronal coupling. To remove all potentially spurious connections of CFS, we discarded observations of interareal CFS between such sources that were also connected by both interareal 1:1 PS and local CFC by using the triangle motif analysis as described above (Fig 1; see also Methods, Removal of potentially spurious CFC connections). We found that after the pruning of all putatively spurious connections, the mean connection density of CFS and its 95th percentile confidence limit remained above zero for CFS for α:β CFS in SEEG and MEG and for δ:α CFS in SEEG (Fig 4A and 4B). In SEEG, the correction removed a larger fraction of CFS connections than in MEG, indicating that the larger initial connection density *K* in SEEG may have been due to putative spurious couplings. In MEG, observations of CFS between γ and Hγ were reduced to near zero, suggesting that this phenomenon was mostly spurious and possibly arising from muscle artifacts [74]. In summary, genuine CFS was observed in human RS brain activity even after application of our correction method, which, because of its conservative nature, likely underestimates the actual number of significant genuine connections.

We next applied the triangle-motif–based correction to remove ambiguous PAC connections, with the difference that amplitude envelopes (S6 Fig) were used instead of PS to detect HF interactions and PAC for detecting local CFC (see Methods, Group-level statistics). After applying the correction method and removing the possibly spurious PAC connections, the connection density for PAC remained significantly above zero (as indicated by the 95% confidence limits) for PAC between θ–α and α–γ oscillations in SEEG as well as for PAC between α and β–γ oscillations in both SEEG and MEG. The connection density values for PAC between γ and Hγ in MEG remained significantly above zero after removing the possibly spurious connections, although they too were greatly attenuated.

Because our correction method for spurious interactions is based on estimation of PS, it is affected by the metric of PS used. For the results presented so far, we used the weighted phase-lag index (wPLI) [75], which yields PS estimates that are not inflated by volume conduction (SEEG) and source leakage (MEG). However, it is insensitive to genuine zero-lag neuronal couplings and may thus underestimate the genuine extent of PS. To test whether this is a significant confounder, we also used the phase-locking value (PLV) to estimate PS. PLV is not markedly sensitive to variation in phase difference, but it is inflated by linear mixing [76]. To compensate for this and reduce the effects of linear mixing, we excluded the signal-leakage–dominated short-range connections from analyses of MEG data (see Methods, Removal of low-fidelity parcels and connections from MEG connectivity analysis). Even so, we found a greater connection density for PS measured with PLV than with wPLI in MEG (S4 Fig). Correspondingly, the correction led to a greater reduction of *K* in MEG CFS (S7 Fig), but importantly, the connection density of α:β CFS remained significantly above zero. In SEEG, the corrected *K* values for PS were more similar between PLV and wPLI, in line with the fact that in appropriately referenced SEEG, volume conduction is well controlled [6]. We also computed corrected PAC values using PLV as the PS metric. Results were largely similar when PLV instead of wPLI was used for estimating LF PS (S7C and S7D Fig). Taken together, these results show that using our novel, to our knowledge, method for removing potentially spurious interareal CFC, genuine interareal CFS and PAC between separable sources can be observed in both SEEG and MEG data and that our method is not qualitatively confounded by the method used for estimating within-frequency PS.

### RS CFS in eyes-open and closed conditions

In the SEEG RS data set used here, participants had their eyes closed to limit the disturbances typical to the clinical environment, whereas eyes-open RS data were acquired from MEG participants for compatibility with visual tasks. Because the amplitude of local α oscillations is greater in the eyes-closed than in the eyes-open state [14, 18, 77], we asked whether the larger *K* values found in SEEG compared to MEG could be explained by differences in the brain state. We recorded new MEG eyes-open and eyes-closed RS data from 10 healthy subjects and computed interareal CFS in the same manner as described above. Significant and qualitatively identical 1:2 CFS between α and β oscillations was observed in both the eyes-open and eyes-closed RS, but with greater *K* values in the eyes-closed than in the eyes-open condition (S8 Fig). This parallels the overall larger *K* values in SEEG data and overall shows that RS CFS is qualitatively unaffected by the RS condition. Because no θ:α CFS coupling was observed in eyes-closed MEG, the θ:α CFS observed in eyes-closed SEEG does not result from the lack of visual input but is a genuine property of the mesoscale brain dynamics observed with SEEG.

### Interareal CFC decreases as a function of distance

**Fig 5:**
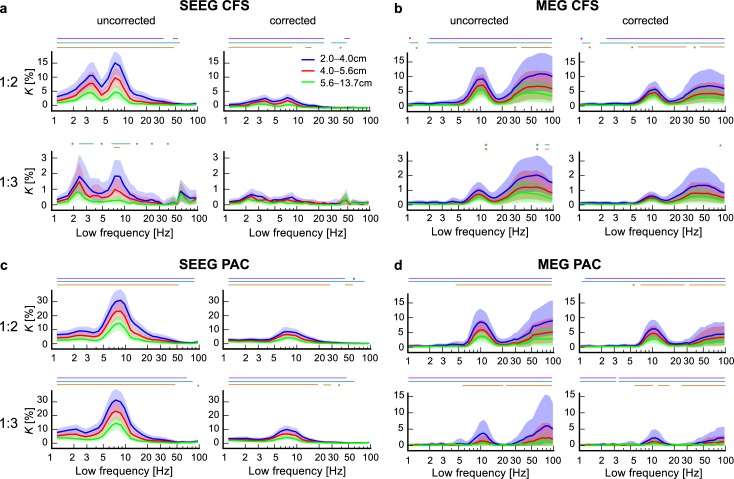
Interareal CFS and PAC decrease as a function of distance. (a) Connection density (*K*) for uncorrected (left) and corrected (right) interareal CFS estimated separately in 3 distance bins containing equal numbers of connections for 1:2 (top row) and 1:3 (bottom row) interareal CFS in SEEG data. All values are plotted, with 95% confidence limits indicated by shades. The colored bars and stars indicate LFs where *K* values between distance bins were significantly different (Wilcoxon test, *p* < 0.05, corrected) between distance bins (purple: short versus medium; turquoise: short versus long; orange: medium versus long). (b) Same as (a) for CFS in MEG. (c) Same as (a) for interareal PAC in SEEG and (d) in MEG. Connection density of CFC was larger for the shortest than for the longest distance bin for CFS at ratio 1:2 and for PAC at ratios 1:2 and 1:3 in both SEEG and in MEG data across most of the frequency spectrum. Plot data and underlying connectome data are available online at https://datadryad.org/stash/dataset/doi:10.5061/dryad.0k86k80. CFC, cross-frequency coupling; CFS, cross-frequency PS; LF, low frequency; MEG, magnetoencephalography; PAC, phase–amplitude coupling; PS, phase synchrony; SEEG, stereoelectroencephalography.

Observations of significant CFC after removal of putative false-positive CFC using a method that minimizes false positives strongly suggests that genuine interareal CFC characterizes human RS brain dynamics. We next set out to investigate whether CFC would be dependent on the distance between the cortical sources. Since prior studies [6, 31, 39, 78] have shown that within-frequency PS is negatively correlated with distance, we expected similar results for CFC. We divided the electrode contact pairs in SEEG and parcel pairs in MEG into 3 distance bins containing equal numbers of connections each and computed 1:2 and 1:3 CFS and PAC, as well as PS, in each of these bins (see Methods, Computation of CFC in distance bins). CFS and PAC were observed in all distance bins in both SEEG and MEG data (Fig 5). For nearly all low frequencies, the greatest *K* values for CFC were found for the shortest distances (blue lines) and the smallest *K* values for the longest distances (green lines) both before and after removing possibly spurious connections, and all 3 bins were found to be significantly different pairwise from each other (Wilcoxon test, *p* < 0.05, corrected for multiple comparisons) for 1:2 CFS and 1:2–1:3 PAC. PS GS also was found to decrease with distance (S3 Fig) as observed before [6, 31, 39, 78].

### Interareal CFC is dependent on laminar depth

**Fig 6:**
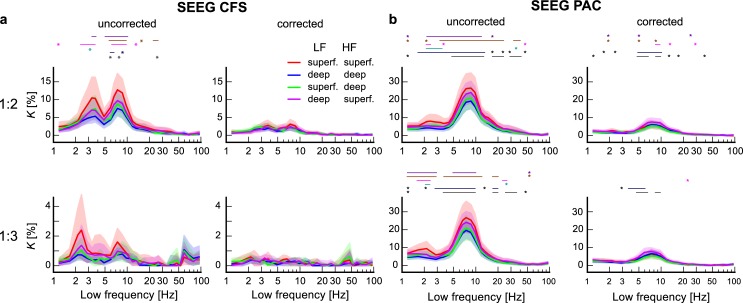
CFS and PAC in different laminar depths in SEEG data. (a) Connection density *K* for uncorrected (left) and corrected (right) interareal CFS at ratio 1:2 (top row) and 1:3 (bottom row) in SEEG among electrode pairs that were either both in more superficial (s) layers (red) or both in deeper (d) layers (blue), when the LF electrode was in a more superficial layer and the HF electrode in a deeper layer (green), and vice versa (purple). The colored bars and stars indicate LFs at which *K* values were significantly different between laminar depth combinations (Wilcoxon test, *p* < 0.05, corrected) (purple: s–s versus d–d; beige: s–s versus s–d; pink: s–s versus d–s; turquoise: d–d versus s–d; dark blue: d–d versus d–s; gray: s–d versus d–s). (b) Same for interareal PAC in SEEG data. In both CFS and PAC, for the uncorrected data, the connection densities were highest for connections in which both electrodes were localized within superficial layers and lowest for both localized within deeper layers. In corrected PAC, *K* was highest when LF electrodes were localized to deeper and HF to superficial layers and lowest when vice versa. Plot data and underlying connectome data are available online at https://datadryad.org/stash/dataset/doi:10.5061/dryad.0k86k80. CFS, cross-frequency PS; HF, high frequency; LF, low frequency; PAC, phase–amplitude coupling; PS, phase synchrony; SEEG, stereoelectroencephalography.

We then investigated whether interareal 1:2 and 1:3 CFS and PAC in SEEG would vary along cortical depth, which would yield insight into the underlying cortical current generators of human CFC interactions. The electrode segmentation algorithm used here enables the separation of electrode contacts in deep and superficial cortical layers (see Methods, Estimation of CFC in distinct cortical layers in SEEG data), but not localization to specific layers because of the electrode size and limits of localization accuracy. Using this approach, a previous study has identified cortical-depth–dependent coupling profiles for within-frequency PS [6]. We found that before the pruning of spurious connections, CFS and PAC of δ–θ and θ–α LF oscillations with higher frequencies showed the largest *K* values between the electrodes in superficial cortical layers (Fig 6, red lines) and the lowest *K* values between those in the deeper layers (blue lines), this difference in *K* being significant for θ:α and α:β CFS at ratio 1:2 and for PAC at 1:2–1:3 over a wide LF range (Wilcoxon test, *p* < 0.05, corrected for multiple comparisons). Values for CFS connections between electrodes in more superficial and deeper layers (green and purple lines) lay between these values. After the pruning of possibly spurious connections, however, these differences were less pronounced and did not exceed the significance threshold. For PAC, connections with the LF electrode in a deeper and the HF electrode in a more superficial layer were now most prominent and those with the inverse relationship least prominent, which was significant over α:β PAC. Thus, while CFC was indeed dependent on cortical depth, further studies are needed to clarify its source in the cortical laminae with more precision, as well as its dependence on interareal PS and local CFC in the correction approach.

We then asked whether MEG could be preferentially sensitive to CFC interactions from either the deeper or more superficial layers. To this end, we measured with parcel degree how central each parcel was in the CFC networks and estimated the correlation between these degrees in MEG and in each of the 4 possible laminar depth combinations in SEEG data (Spearman’s rank correlation test) for α:β and α:γ CFS and PAC (see Methods, Correlation of CFC in MEG with laminar depth in SEEG data). Parcel degree values of α:β CFS in MEG data were positively correlated with degree values when both electrodes were localized in deeper layers and negatively when they were both localized in more superficial layers (S9 Fig, *p* < 0.05, corrected with Benjamini–Hochberg). The difference between the correlation values *r* between different layer combinations was determined to be significant at *z* > 1.96 with a Fisher z-transform for 1:2 CFS (indicated by black bar in S9 Fig). Also, for α:β and α:γ PAC, the parcel degree values of MEG data were positively correlated when both electrodes were localized in deeper layers. These findings thus extend to CFC the notion that MEG may be most sensitive to neuronal current sources in deep cortical layers [79].

### LF and HF hubs differ between CFS and PAC

**Fig 7:**
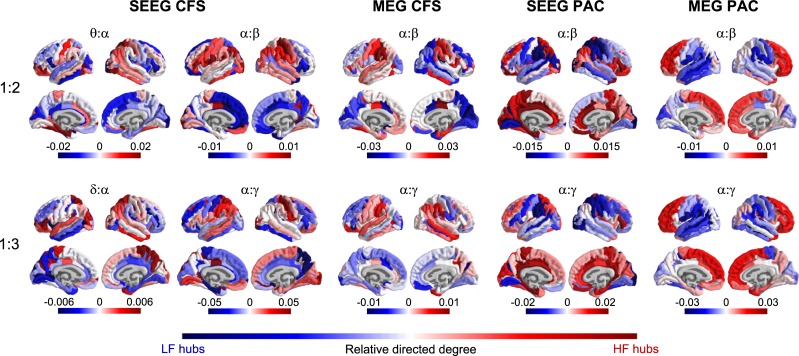
CFC networks have an asymmetric LF and HF hub architecture. The functional organization of CFC networks as measured with localization of LF and HF hubs. Hubness was measured as relative LF and HF degree of each brain region (parcel). Relative degree values indicate whether a parcel is primarily a LF hub (blue) or HF hub (red) in interareal CFC. Top row: brain anatomy of LF and HF hubs for CFS and PAC at ratio 1:2 connecting θ:α and α:β frequencies. Bottom row: brain anatomy of LF and HF hubs for CFS and PAC networks at ratio 1:3 connecting δ:α and α:γ frequencies. CFS and PAC networks show saliently opposing anatomical structures connecting anterior and posterior brain regions. Plot data and underlying connectome data are available online at https://datadryad.org/stash/dataset/doi:10.5061/dryad.0k86k80. CFC, cross-frequency coupling; CFS, cross-frequency PS; HF, high frequency; LF, low frequency; MEG, magnetoencephalography; PAC, phase–amplitude coupling; PS, phase synchrony; SEEG, stereoelectroencephalography.

Finally, we aimed to elucidate the anatomical–topological organization of the δ–θ:α, α:β, and α:γ CFS and PAC connectomes. We first used a conventional in- and out-degree–based graph-theoretical approach [80] to estimate LF and HF centrality across the cortical surface (see Methods, Estimation of functional organization of CFC networks). We represented both SEEG and MEG CFC connectomes in the 148-parcel Destrieux atlas and estimated relative directed degrees. This thus yielded, for each of the main LF peaks, a measure of whether a given parcel was predominantly an HF or an LF hub in the CFC network. For CFS networks, HF β and γ hubs (red) were largest in SM regions, posterior parietal cortex (PPC), and temporal cortex (Fig 7). LF α hubs (blue) were localized to the lateral prefrontal cortex (lPFC) and medial parietal cortex (MPC) in both SEEG and MEG, and in MEG also to the occipital cortex. Hub localization of θ:α and δ:α CFS (which was only observed in SEEG) was similar to that of α:β CFS. However, for PAC, we observed largely an opposite localization of LF and HF hubs in most cortical regions. We found the LF α hubs to be consistently localized to the SM, PPC, and occipitotemporal regions in both SEEG and MEG, and the HF β and γ hubs mainly to be localized to frontal regions and the MPC. In order to confirm the similarity between SEEG and MEG data and the dissimilarity between CFS and PAC, we computed the correlation of relative directed degree values across parcels using Spearman test. A significant (*p* < 0.05) positive correlation between SEEG and MEG was found for α:β and α:β PAC and a nonsignificant one for α:β CFS (Table 1). Relative degree values for α:β CFS and PAC were indeed significantly anticorrelated both in SEEG and MEG (*p* < 0.05) and also for α:γ CFS and PAC in SEEG, but not in MEG (Table 2).

**Table 1:** Parcel values are correlated between SEEG and MEG data.

	Relative Directional Degree		Directionality	
	*r*	*p*	*r*	*p*
1:2 CFS	0.136	0.101	0.094	0.254
1:2 PAC	**0.233**	**0.004**	**0.259**	**0.002**
1:3 CFS	−0.052	0.531	0.130	0.114
1:3 PAC	**0.203**	**0.013**	**0.314**	**<10**^**−4**^

Values obtained with Spearman test. Significant correlations (*p* < 0.05) in bold. **Abbreviations:** CFS, cross-frequency PS; MEG, magnetoencephalography; PAC, phase–amplitude coupling; PS, phase synchrony; SEEG, stereoelectroencephalography.

**Table 2:** Parcel values are anticorrelated between CFS and PAC.

	Relative Directional Degree		Directionality	
	*r*	*p*	*r*	*p*
1:2 SEEG	**−0.254**	**0.002**	**−0.277**	**0.001**
1:2 MEG	−0.114	0.168	−0.106	0.200
1:3 SEEG	**−0.241**	**0.003**	0.018	0.831
1:3 MEG	**−0.240**	**0.003**	**−0.252**	**0.002**

Values obtained with Spearman test. Significant correlations (*p* < 0.05) in bold. **Abbreviations:** CFS, cross-frequency PS; MEG, magnetoencephalography; PAC, phase–amplitude coupling; PS, phase synchrony; SEEG, stereoelectroencephalography.

To corroborate this graph-theoretical, parcel-degree–based approach, we then directly estimated the preferential directionality of each CFC connection between parcels and, by pooling these connections, asked whether in CFC connections, one parcel predominantly was the location of either the LF or HF oscillation. We estimated such low-versus-high directionality for each parcel pair and each frequency pair of the main peaks across subjects (see Methods, Estimation of functional organization of CFC networks). Significant directionality between the 2 parcels was established if the absolute directionality was higher than in 95% of permutations. We then averaged for each parcel the significant directionality values, again yielding a positive value for parcels that are predominantly HF hubs and a negative value for parcels that are predominantly LF hubs. The results were remarkably similar to those of the degree-based hubness analysis and also corroborated the salient dissociation in the directionality between CFS and PAC (S10 Fig). Estimation of similarity between SEEG and MEG and the dissimilarity between CFS and PAC using the Spearman test yielded results similar to those we obtained for the directed degree (Tables 1 and 2). Taken together, these results provide strong evidence that the anatomy and structure of CFS and PAC connectomes are distinct.

### RS CFS predicts performance in neuropsychological tests

**Fig 8:**
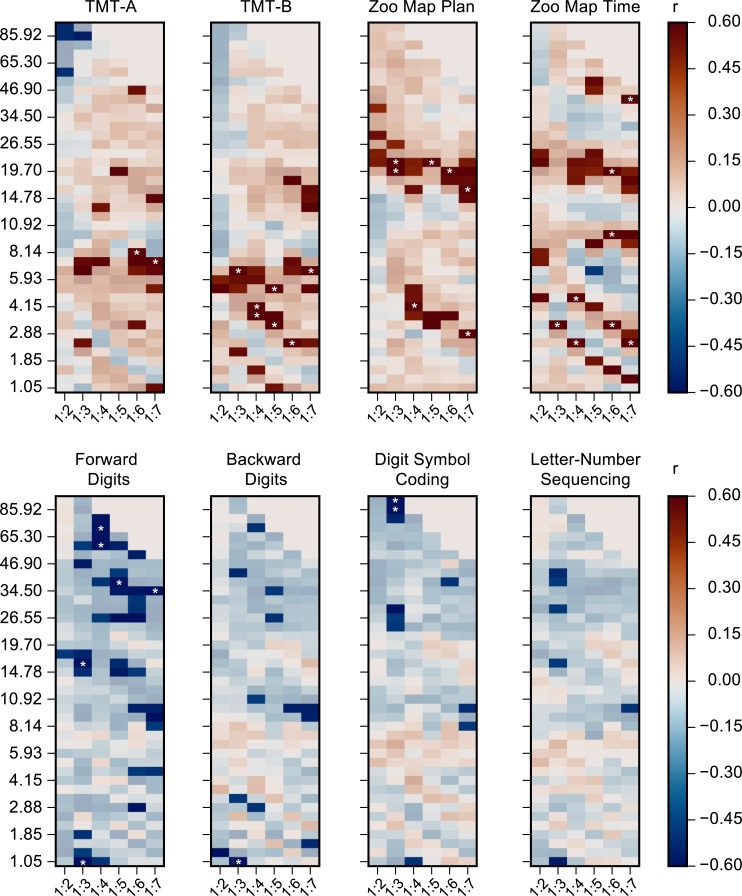
Correlation of CFS with neuropsychological test scores. The correlation of CFS GS in MEG data with scores from neuropsychological assessments for all ratios and frequency pairs (Spearman rank correlation test, *p* < 0.05). The assessments include TMTs A and B and Zoo Map Plan and Time Tests, as well as Forward Digits, Backward Digits, and Digit Symbol Tests, and the Letter-Number Sequencing from Wechsler Adult Intelligence Scale–III. Red color indicates a positive correlation, so that stronger CFS is associated with better performance, while blue indicates a negative correlation between CFS and performance. Correlations with *p* > 0.05 are masked (low saturation colors). The asterisks indicate the observations that remain significant after correction for multiple comparisons (see Methods, Neuropsychological assessment and correlation of CFC with neuropsychological test results) across the 8 neuropsychological tests and CFC frequency pairs. Plot data and underlying connectome data and neuropsychological data are available online at https://datadryad.org/stash/dataset/doi:10.5061/dryad.0k86k80. CFC, cross-frequency coupling; CFS, cross-frequency phase synchrony; GS, graph strength; MEG, magnetoencephalography; TMT, Trail-Making Test.

If CFC is a neuronal coupling mechanism that enables the integration of processing distributed to functionally specialized frequency bands [9, 33–37, 39, 42, 57], its recruitment upon task demands may be limited by individual factors in a trait-like fashion. In this case, RS CFC could be predictive of individual performance in complex cognitive tasks that demand extensive functional integration. To investigate whether the RS CFC networks identified in the prior analyses were predictive of cognitive task performance, we estimated the correlation of CFS and PAC individual GS in RS MEG data with neuropsychological test scores collected separately (Methods, Neuropsychological assessment and correlation of CFC with neuropsychological test results). We computed the correlation of the test scores with CFS or PAC GS separately for each LF and for each frequency ratio (Spearman rank test). CFS between θ–α with β–γ oscillations (θ–α: β–γ CFS) and CFS between β and γ oscillations (β:γ CFS) showed significant positive correlations with scores on Trail-Making Tests (TMTs), which measure visual attention, speed of processing, and central executive functions, as well as with Zoo Map Tests, which measure planning ability (*p* < 0.05, Spearman rank correlation test, Fig 8). Intriguingly, negative correlations with the test scores were observed for CFS of α and β oscillations with higher frequencies (α–β:γ) and for γ:Hγ CFS in the Digits Tests measuring WM performance. In contrast to CFS, PAC was largely uncorrelated with performance in any of these tests, although γ:Hγ PAC was negatively correlated with performance in TMT-A (S11 Fig). For all tests together, PAC did not exceed the threshold for significance. These results suggest that in a trait-like manner, individual RS CFC brain dynamics are predictive of the variability in behavioral performance in separately measured tasks, which supports the notion that CFC plays a key functional role in the integration of spectrally distributed brain dynamics to support high-level cognitive functions.

## Discussion

Several CFC mechanisms, especially PAC [9, 34–37] and cross-frequency phase synchrony (CFS [9, 38–41], have been proposed to coordinate neuronal processing across multiple frequencies and regulate the communication among coupled oscillatory networks over frequencies. Networks of phase-coupled oscillations distributed across brain areas and multiple frequencies are a core systems-level mechanism for cognition functions [11, 25–27]. Yet, only a few studies have identified CFC in cortex-wide networks and across multiple frequency pairs [39, 41, 56, 60]. We propose that that cortex-wide CFC networks are essential for coordinating computations across many frequencies and across multiple brain regions concurrently to support complex brain dynamics and cognitive functions. We report the presence and organization of 2 forms of interareal CFC, CFS and PAC, in human SEEG and MEG RS brain activity. Importantly, the validity of all prior CFC findings has also come into question because filtering artifacts arising from nonsinusoidal signals lead to spurious observations of CFC. Here, we used a novel, to our knowledge, graph-theoretical network-motif–based method to distinguish genuine and putatively spurious CFC in large-scale CFC networks. Genuine interareal CFS and PAC both characterized human RS activity but showed distinct spectral profiles, anatomical architectures, and coupling directions across distributed brain regions, which strongly suggests that they originate from distinct neurophysiological mechanisms and play unique functional roles. The strength of CFS networks was also predictive of the behavioral performance in neuropsychological assessments performed separately, implying a trait-like role for individual CFS in complex cognitive tasks. Overall, these data conclusively establish the presence of 2 distinct types of interareal CFC that are in a position to underlie the coordination of neuronal processing across anatomically distributed networks in multiple oscillatory frequencies.

### Large-scale CFC networks characterize human RS brain activity

Human brain activity during rest is characterized by intrinsically correlated fluctuations in networks of brain regions, first identified with fMRI [28, 29]. Also, in human electrophysiological measurements, PS and amplitude correlations of neuronal oscillations characterize RS activity in a wide range of frequencies in anatomically well-delineated structures [3–8, 81] with a modular architecture and colocalized phase and amplitude correlations [31]. It has, however, remained unknown whether RS activity is also characterized by CFC networks and how networks formed by CFC would be organized. We report here the presence of genuine interareal PAC and CFS in large-scale RSNs. PAC of θ and α oscillations with higher frequencies was robust throughout all investigated ratios from 1:2 up to 1:7 in SEEG data and up to 1:4 in MEG data, indicating that the phases of θ and α oscillations were coupled with the amplitudes of β, γ, and Hγ oscillations. PAC has been suggested to reflect the regulation of sensory information processing achieved in higher frequencies through excitability fluctuations imposed by slower oscillations [9, 10, 34, 36, 37, 42]. These results, despite methodological differences, are similar to previous findings that have reported PAC in the rat hippocampus [46, 47], nonhuman primates [82, 83], and in human intracranial EEG [48–51, 60] and EEG and MEG recordings [54–56, 84–86]. In contrast to PAC, interareal CFS connected the phase of θ/α oscillations with the phases of β and γ oscillations only with small frequency ratios (1:2 and 1:3). CFS, by definition, reflects a stable phase difference between coupled oscillations and thus is, by definition, associated with consistent spike–time relationships between the neuronal assemblies in the 2 CFS-locked frequency bands [57]. The lack of high frequency ratios in CFS is not surprising because CFS necessitates the slow oscillation having a temporal accuracy in the subcycle timescales of the fast oscillation. Hence, stable phase differences over large frequency ratios may be limited by the temporal accuracy in the cellular, synaptic, and circuit mechanisms generating the slower oscillations. However, transient CFS at larger ratios has been observed during task performance [41].

CFS and PAC similarly were observed in both SEEG and MEG data, indicating that their interareal CFC characterizes both the meso- (mm) and macro- (cm) scale brain dynamics. Pronounced local α oscillations have been recognized as a marker of RS human brain activity for several decades [18, 77]. Here, we show that these α oscillations are systematically cross-frequency–coupled with the faster β- and γ-frequency oscillations across long distances in both SEEG and MEG. CFC was present in both eyes-open and eyes-closed brain states, albeit stronger in eyes-closed data, putatively contributing to larger CFC values in SEEG in addition to better signal/noise ratio.

### Characteristics of CFC networks in RS human brain activity

A central unresolved question in research aimed at emergent brain dynamics is whether networks of interareal CFC characterize resting human brain activity with consistent large-scale architectures. Prior studies have found phase synchronization within frequencies in SEEG [6] and in MEG data [31, 39, 78] to decrease as a function of distance. In this study, we found that the connection density of interareal CFS and PAC decreased with distance between electrode contacts (SEEG) or cortical parcels (MEG), similarly to that of interareal PS.

Intracortical recordings with laminar probes have shown that oscillations of different frequencies are generated differentially across cortical layers [87–90]. We identified the depth in cortical gray matter of each SEEG electrode [6, 91] and estimated the strength of CFC within and between cortical depths. Before the exclusion of potentially spurious connections, CFS and PAC were significantly stronger among electrode pairs that were both located in more superficial layers than between pairs both located in deeper layers. However, possibly because of a decreased number of samples in corrected data and perhaps because a larger proportion of superficial connections were putatively spurious, differences between depths were not significant for CFS after correction. For PAC, we found the greatest connection density values for connections in which the slow θ–α oscillations were located in the deeper layers and the fast β oscillations in the more superficial layers, and conversely, the lowest values for those in which it was vice versa. This parallels earlier studies, which showed that γ synchrony is strongest in superficial layers 2–3, whereas α oscillations and synchrony are generally more pronounced in the deeper layers in both monkeys and mice, although this varies somewhat with the cortical regions [87–90]. It is important, however, to note that without a current source density analysis, for which the SEEG electrode contacts and their separation are too large, and because of complex current source geometries in cortical circuitry and the volume conduction between layers [91], our findings should be corroborated and expanded in future studies addressing the neuronal sources of CFC in different cortical layers with appropriate laminar probes.

Interestingly, our data showed that CFC in MEG, in terms of the cortical node-degree structure, is most similar with that observed with SEEG when both electrode contacts were localized into the deeper layers. This result strongly suggests that the sensitivity of MEG to the postsynaptic currents in large, asymmetric, and well co-oriented neurons, i.e., the pyramidal neurons in cortical layers 5 and 6 that are central in thalamocortical loops [79, 92, 93], biases the detection of CFC with MEG towards the postsynaptic currents in these neurons.

### Distinct large-scale organization of directional network architecture of CFS and PAC

To assess the large-scale architecture of the cortical CFC networks, we used 2 strategies for identifying the hubs of LF α and HF β and γ oscillations. These hubs are the brain regions where predominantly the slower (α) or the faster oscillations (β and γ) of the CFC connection are observed. We found that the LF α and HF β and γ hubs and their directional interactions were asymmetrically localized between anterior and posterior brain regions. The localization of hubs was largely similar between SEEG and MEG data, which corroborates the validity of these findings. Importantly, however, we observed distinct and partially opposing localization of the LF and HF hubs for CFS and PAC. In α:β and α:γ CFS, the α LF hubs were observed in PFC and medial regions that belong to the default mode network [29] or to control and salience networks in the functional parcellation based on fMRI BOLD signal fluctuations [94–96]. This is line with many previous studies that have found α oscillations in these regions to be correlated with attentional and executive functions [14–19]. In contrast, the β and γ HF hubs were found in more posterior regions such as the SM region and the occipital and temporal cortices, where β and γ oscillations are often associated with sensory processing [15, 20–22].

In contrast to CFS, the α LF hubs of PAC were found in the occipital, temporal, and PPC, whereas the β and γ HF hubs were found in the PFC and MPC. This anatomical structure between LF and HF hubs was essentially opposite to the anatomical structure observed for CFS. These results imply that both CFS and PAC contribute to the coordination of intrinsic/task-negative and extrinsic/task-positive RS networks [13, 28, 97] but at least partially with opposite directional roles.

### RS CFS networks predict individual cognitive variability

If CFC regulates communication between within-frequency oscillatory networks to support integration of separate computational functions of cognition [9, 33–37, 39, 42, 57], it should be correlated with the psychophysical performance in cognitive tasks. We have previously shown that α-, β-, and γ-band oscillations and PS networks in these frequencies are coupled via CFS and predict performance in a visual WM task. These observations imply that CFS could coordinate the representation of sensory information achieved in γ frequencies with the executive control achieved in the α band [41]. Because within-frequency PS networks during RS have been shown to form a core underlying the task-state networks [8, 32], we hypothesized here that CFC RS networks might thus also be predictive of cognitive performance in a trait-like manner. We thus estimated the correlation of CFS and PAC GSs in MEG data with the individual variability in cognitive performance in an array of neuropsychological tests. The CFS network strength was indeed predictive of test performance. CFS of θ–α oscillations with β–γ band oscillations and CFS between β and γ oscillations were correlated positively with performance in TMT-A and TMT-B as well as with the Zoo Map Time Test, which measure the interplay of visual processing speed and central executive functions. Negative correlations were found between the strength of CFS and digit test measuring WM performance. These results thus suggest that RS CFS networks indeed predict individual cognitive capacities in a trait-like manner.

### CFS and PAC are distinct CFC mechanisms

Many neurophysiological models have been developed to explain how the phase of slow oscillations reflecting fluctuations in neuronal excitability can regulate the power of fast oscillations, usually in the γ-frequency band via PAC [9, 33–36, 98]. PAC, by definition, is unrelated to spike synchronization between the slow and fast oscillations per se and reflects either the regulation of fast neuronal processing regulated by slower excitability fluctuations or the entrainment of slower oscillations by intermittent bursts of fast oscillations. We have postulated that CFS may support different computational functions than PAC [57]. CFS is a form of PS in which the stable phase difference takes place between 2 neuronal assemblies that oscillate with an m:n frequency ratio [38, 39, 41]. Therefore, the coupling of the phases of the faster and slower oscillations indicates, by definition, that CFS will be associated with consistent spike–time relationships between the neuronal assemblies in different frequency bands. For example, for the α:β CFS, the spikes locked to the beta oscillation would have a consistent spike–time relationship, with spikes locked to the alpha oscillation in every second β cycle. In the current study, spectral and ratio differences, distinct large-scale anatomical structures, and directionalities of CFS and PAC, as well as the differential correlation of the connectomes with the scores of neuropsychological assessments, provide evidence that CFS and PAC impose distinct computational functions and likely arise via separable neurophysiological mechanisms. This conclusion is in line with prior findings during task performance in which we found interareal CFS and PAC to show distinct spectral profiles and CFS, but not PAC, to predict WM performance [41]. Together, these results strongly suggest that CFS and PAC are not simply different operationalizations of a shared CFC process, but rather mechanistically, phenomenologically, and functionally distinct CF coupling mechanisms.

### Genuine positive interareal CFC is not explained by spurious connections

The main goal of this study was to investigate whether genuine interareal CFC characterizes SEEG and MEG data during RS. Our study was motivated by the multiple concerns that have been raised about the validity of previous observations of CFC [61–69]. To map genuine large-scale networks from human SEEG and source-reconstructed MEG data, we introduced a novel, to our knowledge, graph-theory–based approach to identify and exclude all putatively spurious CFC interactions. We first verified the validity of this approach using simulations in coupled Kuramoto oscillators, and then used it to control for possibly spurious CFC connections in SEEG and MEG data. Because our approach may also discard a subset of genuine CFC connections, it gives a conservative lower-bound estimate of the presence of genuine neuronal interareal CFC. The number of significant CFC connections was indeed reduced by the exclusion of the potentially spurious corrections, indicating that part of the commonly observed CFC in SEEG and MEG data may be caused by filter artifacts arising from nonsinusoidal signal components [61–68] or by amplitude fluctuations of non-zero–mean waveforms [69]. However, our results clearly also indicate that genuine neuronal CFC characterizes SEEG and MEG data.

Our method was based on assessing, for each observation of interareal CFC between areas A and B, whether there is also observed interareal within-frequency PS and local CFC that together may lead to a spurious observation of interareal CFC. Because our method uses within-frequency PS to identify the putatively spurious connections, the number of identified potentially spurious connections depends on the PS metrics. Especially in MEG, PLV connection density values were larger than the corresponding wPLI values because PLV is inflated by volume conduction and source leakage [10, 76, 99], whereas wPLI [75] is insensitive to all linear mixing, including also true zero-lag phase coupling. Consequently, use of PLV as a PS metric led to a larger reduction of CFS in MEG than correction with wPLI, but importantly, significant genuine CFC was observed with both methods. While in this study, we focused on identifying genuine interareal CFC in continuous RS data, this approach is adaptable to analyses of event-related data and may thus be used to assess the presence of genuine interareal CFC during task performance.

### Conclusions

We show here that large-scale networks of genuine neuronal CFS and PAC characterize human RS brain activity in SEEG and MEG data. Using a new graph-theoretic approach, we eliminated observations of interareal CFC that could be explainable by filter artifacts. The directional organization of CFC networks showed that CFC coupled slow and fast oscillations between anterior and posterior parts of the brain, suggesting that RS CFC coordinates intrinsic and extrinsic processing modes. The strength of CFS networks was also predictive of cognitive performance in a separate neuropsychological assessment, which implies that individual CFS is a functionally significant, trait-like property of spontaneous brain dynamics. Salient differences in spectral patterns, functional organization, and behavioral correlates demonstrated that CFS and PAC are phenomenologically and functionally distinct and thus likely to serve complementary computational functions. Altogether, converging results from SEEG and MEG data provide strong evidence for the coexistence of 2 forms of genuine neuronal interareal CFC in human RS brain activity and reveal their large-scale network organization.

## Methods

### Ethics statement

All research was carried out according to the Declaration of Helsinki. Prior to the study, each subject signed an informed and written consent. The study protocol for SEEG, computerized tomography (CT), and MRI data obtained in the La Niguarda Hospital were approved by the ethical committee of the Niguarda “Ca Granda” Hospital, Milan (ID 939). The study protocol for MEG and MRI data obtained in the University of Helsinki was approved by the Coordinating Ethical Committee of Helsinki University Central Hospital (HUCH) (ID 290/13/03/2013).

### Modeling

We used a Kuramoto model [71] to investigate the direct and indirect effects of within- and cross-frequency phase coupling on phase correlations observable among neuronal populations. The model was adapted from conventional Kuramoto models so that it comprised 2 “areas” that each contained 2 populations (*N* = 500) of oscillators; one at LF and another at HF so that their frequency ratio was 1:2. The model was defined so that for each area *k*, *k* = 1, …, 4, the phase of each oscillator *h*, *h* = 1, …, 500, was given by

$$\frac{d\theta_{h,k}}{dt}=\omega_{h}+\sum_{\begin{array}{c} k=1\\ l\neq k \end{array}}^{4}\sum_{\begin{array}{c} h=1\\ j\neq i \end{array}}^{500}\kappa_{internal}sin(\theta_{j,k}-\theta_{h,k})+\kappa_{external,k,l}M_{l}sin(\theta_{l}-\theta_{h,k}),$$

where the phase increment per sample *ω*_*i*_ was uniformly distributed in the range from π/*m* to π/15*m* with *m* = 1 for HF and *m* = 2 for LF. Oscillators within the populations were all-to-all connected with constant weak coupling *κ*_*internal*_ = 0.12. The populations were 1:1 (*ε*_LF PS_ and *ε*_HF PS_) or 1:2 (*ε*_Local CFS_ and *ε*_Interareal CFS_) phase coupled with oscillators of the other populations (see Fig 2A) so that the coupling was mediated by the population mean signals *M*_*k*_, $M_{k}=\frac{1}{N}|\sum_{h=1}^{N=500}e^{i\theta_{h}}|$. The coupling between the populations, *κ*_*external*_, was varied with a shared coupling factor, *c*, so that *κ*_*external*_ = *cε* for each connection specified by the corresponding *ε* value.

To validate our method for correction of spurious CFS, we set *ε*_interareal CFS_ = 0, *ε*_Local CFS_ = 0.3, and *ε*_LF PS_ = *ε*_HF PS_ = 0.5 in order to produce model time series that had no genuine interareal CFS, but rather only spurious interareal CFS that emerges indirectly from the combination of interareal PS and local CFS. We performed significance tests for PS and both forms of CFS. We simulated 100,000 iterations of the model, yielding 5,000 cycles at HF (at nominal *ω*_*i*_ = *π*/10) across 512 values of *c* from 0 to 0.3. Observations of PS and CFS were deemed significant at a nominal *p* < 0.01, obtained by setting the threshold for the observed PLV to 2.42 times the null-hypothesis PLV.

### SEEG data acquisition

SEEG data were recorded from 59 subjects affected by drug-resistant focal epilepsy and undergoing presurgical clinical assessment at “Claudio Munari” Epilepsy Surgery Centre, Niguarda Hospital, Milan. Intracranial “monopolar” (i.e., all contacts referenced to a single contact in the white matter) LFPs were recorded with platinum–iridium, multilead electrodes with 8–15 contacts each. These contacts were 2 mm long, 0.8 mm thick, and had an intercontact border-to-border distance of 1.5 mm (DIXI medical, Besancon, France). The neuroanatomical targets and numbers of electrodes implanted to each subject varied exclusively according to clinical requirements [100].

For each subject, one 10-minute set of eyes-closed RS data was recorded with a 192-channel SEEG amplifier system (NIHON-KOHDEN NEUROFAX-110, Tokyo, Japan) at a sampling rate of 1,000 Hz. The electrode contact positions were localized after the implantation by using CT scans and the SEEGA tool, which performs automatic electrode contact localization and is freely available [91, 101]. Structural MRIs were recorded before implantation, and rigid-body coregistration was used to colocalize MRIs and postimplant CT scans [100, 102]. Based on this, electrode contacts were assigned to one of 148 parcels of the Destrieux atlas [73].

### SEEG data preprocessing and filtering

Defective electrode contacts were identified by nonphysiological activity and excluded from further analysis. For referencing, we used the closest-white–matter referencing scheme [102], in which each contact in cortical gray matter is referenced to the nearest contact in white matter. The seizure-onset and propagation zones were identified by clinical experts in gold-standard visual analysis, and contacts in these areas were excluded from analysis, as were contacts from subcortical regions. In order to avoid spurious connectivity due to volume conduction, we excluded from connectivity analyses also contact pairs that shared the same reference or had a contact-to-contact distance < 2 cm.

We excluded all harmonics of 50-Hz line noise using a band-stop equiripple finite-impulse–response (FIR) filter. Moreover, because interictal epileptic events (IIEs) such as interictal spikes are characterized by high-amplitude fast temporal dynamics as well as by widespread anatomical spread, filtering artifacts may occur around epileptic spikes and artificially inflate both PS and CFC estimates. We therefore discarded periods containing IIEs so that we first divided the signal in nonoverlapping 500-ms time windows and detected IIEs in amplitude envelopes by values that exceeded the channel mean amplitude > 5 standard deviations. Time windows during which at least 10% of cortical contacts demonstrated IIEs in more than half of the 18 frequency bands were then excluded from further analyses. Time series were then filtered with Morlet wavelets with *m* = 5 using 49 roughly logarithmically spaced center frequencies from 1.2 to 315 Hz and downsampled to a sampling rate approximately 5 times greater than the wavelet center frequency.

### MEG and MRI data acquisition

For the main study, 306-channel MEG (204 planar gradiometers and 102 magnetometers) was recorded with a Vectorview/Triux (Elekta-Neuromag/MEGIN, Helsinki, Finland) at the BioMag Laboratory, HUS Medical Imaging Center from 19 healthy participants during 10 minutes of eyes-open RS. Overall, 27 sets of RS MEG data were obtained, with 4 participants contributing 2 sets and 2 participants contributing 3 sets. Subjects were instructed to focus on a cross on the screen in front of them. Bipolar horizontal and vertical EOG were recorded for the detection of ocular artifacts. MEG and EOG were recorded at a 1,000-Hz sampling rate. T1-weighted anatomical MRI scans (MP-RAGE) were obtained for head models and cortical surface reconstruction at a resolution of 1 × 1 × 1 mm with a 1.5-Tesla MRI scanner (Siemens, Munich, Germany) at Helsinki University Central Hospital. Written informed consent was obtained from each subject prior to the experiment. In addition to the main MEG data set, we also recorded a de novo 10-subject cohort (of which 4 had also participated in the main study) with 10-minute sessions of both eyes-open and eyes-closed RS. These data were recorded and preprocessed in a manner identical to the main MEG data set.

### Cortical parcellation

FreeSurfer software (http://surfer.nmr.mgh.harvard.edu/) was used for volumetric segmentation of MRI data, flattening, cortical parcellation, and neuroanatomical labeling with the Destrieux atlas [73]. We obtained a cortical parcellation of 200 parcels by iteratively splitting the largest parcels of the Destrieux atlas along their most elongated axis at the group level [72, 103]. All analyses of MEG data in the main data set were carried out using the 200-parcel parcellation, except the degree and directionality analyses (see Methods, Estimation of functional organization of CFC networks), for which the data were collapsed to the original 148-parcel Destrieux atlas to facilitate the comparison with SEEG data. The analysis of the additional 10-subject MEG data set was carried out in the 148-parcel Destrieux atlas.

### Source models and colocalization

MNE software (https://mne.tools/stable/index.html) [104, 105] was used to create cortically constrained source models, for MEG–MRI colocalization, and for the preparation of the forward and inverse operators. The source models had dipole orientations fixed to pial-surface normals and a 5-mm interdipole separation throughout the cortex, which yielded 5,086–7,857 source vertices per hemisphere.

### MEG data preprocessing and filtering

Temporal signal space separation (tSSS) in the Maxfilter software (Elekta-Neuromag) [106] was used to suppress extracranial noise from MEG sensors and to interpolate bad channels. We used independent components analysis (ICA) adapted from the MATLAB toolbox Fieldtrip, http://www.fieldtriptoolbox.org/, to extract and identify components that were correlated with ocular artifacts (identified using the EOG signal), heartbeat artifacts (identified using the magnetometer signal as a reference), or muscle artifacts. After artifact exclusion, the time-series data were filtered into NB time series using a bank of 53 Morlet filters with wavelet width parameter *m* = 5 and approximately log-linear spacing of center frequencies ranging from 1.1 to 315 Hz. After the filtering, the time-series data were downsampled to a sampling rate around 5 times the center frequency.

### MEG source reconstruction: Inverse transform and collapsing of source signals to parcel time series

We computed noise covariance matrices (NCMs) using preprocessed (see Methods, MEG data preprocessing and filtering) and FIR-filtered (151–249 Hz) MEG RS data time series. NCMs were evaluated in and averaged across 60 time windows of 10 s. This frequency band was used for NCMs because it comprises environmental, sensor, and biological noise components but less neuronal activity than the lower-frequency bands. These NCMs were then used for creating one inverse operator per subject with the MNE software and the dSPM method with regularization parameter λ = 0.11 [104, 105]. In analyses of interareal correlations with source-reconstructed MEG data, one confounder is posed by the spurious connections resulting from source leakage that spreads true interareal into false positives in their vicinity [10, 76]. In order to mitigate these effects and collapse the inverse transformed source-dipole (vertex) time series into parcel time series in a manner that maximizes the source-reconstruction accuracy [107], we used estimates of “vertex fidelity” to obtain fidelity-weighted inverse operators. The fidelity estimates were obtained by simulating for all 200 parcels uncorrelated, complex white-noise time series (equivalent to decimated Morlet-filtered white noise) and then applying these parcel time series to all source dipoles per parcel. The source time series, *Z*_*V*,*orig*_, were then forward and inverse modeled (i.e., considered as ground-truth parcel data, transformed into MEG sensor time series, and then source-reconstructed) to obtain source time series, *Z*_*V*,*mod*_, that thus encompass the effects of MEG-data-acquisition–related signal mixing and residual inverse-modeling source leakage. Then, vertex fidelity was estimated for each source vertex by the correlation between the forward-inverse–modeled data and ground-truth data. This correlation was quantified with the absolute-valued real part of the complex PLV (cPLV) between *Z*_*V*,*orig*_ and *Z*_*V*,*mod*_, which was defined [38, 39, 108] as follows:

$$c{PLV}_{orig,mod}=\frac{1}{N}\sum_{t}\exp\left\{i{(\theta }_{V,orig}-\theta_{V,mod})\right\},$$

where *θ* is the phase of a complex filtered time series *Z*. Each source-dipole row of the inverse operator was then weighted with

$$w=sign\left(re({cPLV}_{orig,mod})\right)∙{\left(re({cPLV}_{orig,mod})\right)}^{2}.$$

The fidelity-weighted inverse operator has higher reconstruction accuracy than a regular inverse operator for the given parcellation because it gives greater weight to sources with better reconstruction accuracy for the signals from the parcels they belong into. The NB RS time series were inverse modeled using this operator and collapsed into the parcellation by simply averaging the sources for each parcel. The sign operation ensures that the source current polarity switching, for example, on opposing walls of a sulcus, does not result in signal cancelation at averaging.

### Removal of low-fidelity parcels and connections from MEG connectivity analysis

To focus further analyses on parcel pairs of which the interaction can be estimated with reasonable accuracy, we measured the quality of the parcellation-collapsed inverse transform with “parcel fidelity” and “cross-parcel mixing.” Similarly to the procedure for estimation of vertex fidelity described in Methods, MEG source reconstruction: Inverse transform and collapsing of source signals to parcel time series, we simulating uncorrelated, complex white-noise time series for all parcels, applied forward and inverse transforms, and collapsed vertex time series into parcel time series using the fidelity-weighted inverse (see Methods, MEG data preprocessing and filtering). We then estimated parcel fidelity as the absolute real part of cPLV between the original and forward-inverse–modeled parcel time series for the 200 parcels concurrently and also estimated cross-parcel mixing among all parcels as the |re(cPLV)| between all forward-inverse–modeled time series [41].

To decrease the probability of spurious synchronization and exclude poorly source reconstructable connections, for the wPLI analysis, we excluded parcels with parcel fidelity < 0.1, retaining 187 of 200 parcels and 34,782 (87.4%) of all 39,800 parcel pairs. For the PLV analysis, which is affected by source leakage, we additionally excluded parcel pairs with cross-parcel mixing of PLV > 0.2143, so that in these analyses, we retained 28,416 (71.4%) of parcel pairs. The threshold for cross-patch PLV was obtained as 1.95 times the mean value in the simulations, which corresponds to a nominal *p* < 0.05 significance level. The removed parcels and connections were located mostly in deep and/or inferior sources, which are known to generate the least detectable signals in MEG and are hence most likely to generate spurious connections [109].

### Analysis of interareal phase synchronization

To identify cortex-wide PS networks, we first computed individual electrode contact-to-contact (SEEG data) or parcel-to-parcel (MEG data) interaction matrices. PS was estimated using the wPLI [75] and the PLV [38, 39]. Because of residual linear mixing between the parcel time series after inverse modeling, i.e., source leakage, the PLV yields inflated values and artificial zero-lag false-positive observations, while wPLI is insensitive to all zero-lag interactions and hence does not yield artificial interactions or true zero-lag couplings [75, 110].

We computed PS across the whole time series for each frequency, and each contact pair *c*_*a*_, *c*_*b*_ or parcel pair *p*_a_, *p*_*b*_ with wPLI and PLV. The wPLI was defined as

$${wPLI}_{a,b}=\frac{\left|E\{im(X_{ab})\}\right|}{E\{\left|im(X_{ab})\right|\}}=\frac{\left|E\{\left|im(X_{ab})|sign(im(X_{ab})\right)\}\right|}{E\{\left|im(X_{ab})\right|\}},$$ (1)

where *im* (*X*_*ab*_) is the imaginary part of the cross spectrum of the complex time series *Z*_*a*_ and *Z*_*b*_ and *E{}* is the expectancy value operator. Here, we substituted the cross spectrum with = $Z_{a}Z_{b}^{*}$, where *Z*_*a*_ and *Z*_*b*_ are Morlet-filtered NB time series and * denotes the complex conjugate, and used the mean over samples as the expectancy value. This can be done because Fourier- and Morlet-based spectral analysis are mathematically equivalent [111].

The PLV was defined as

$${PLV}_{a,b}=\frac{1}{N}|\sum_{t}exp\{i{(\theta }_{a}-\theta_{b})\}|,$$ (2)

where *θ* is the phase of the complex filtered time series *Z* and *N* is the number of samples. To assess the significance of synchronization at the level of individual subjects, we obtained one surrogate PS value for each contact/parcel pair in which *Z*_*b*_ was randomly rotated (shifted by a random number of samples) and then calculated the means (wPLI_surr_mean_, PLV_surr_mean_) and, for wPLI, also standard deviations (wPLI_surr_SD_) of these surrogate PS estimates across contact/parcel pairs. Rotation, rather than more aggressive shuffling methods, was used to retain the autocorrelation structures in the data for the surrogate analyses and thereby avoid the underestimation of the null-hypothesis–level PS values. We then computed for observed wPLI values, wPLI_obs_, the z-score as

$$z=\frac{{wPLI}_{obs}-{wPLI}_{surr\_mean}}{{wPLI}_{SD}}$$

and considered those wPLI_obs_ values significant for which z > 2, corresponding to α = 0.02. The PLV of uncorrelated phases is Rayleigh distributed, and the Rayleigh distribution is only a function of its mean so that the distribution reaches the 99th percentile at PLV = 2.42 PLV_surr_mean_ [39]. We used α = 0.01 as the significance criterion for the measured PLV, PLV_meas_, and thus, connections with PLV_meas_ > 2.42 PLV_surr_mean_ were considered significant. Using this approach, we obtained for each subject and each frequency the individual connection density (*K*) values, where *K* indicates the proportion of significant connections of all possible connections across channel pairs in SEEG and parcel pairs in MEG.

### Analysis of local and interareal of CFC: PAC and CFS

CFS and PAC were computed between all LF and HF frequency pairs at ratios of *n*:*m* (LF:HF) from 1:2 to 1:7, and for each contact pair *c*_*a*_, *c*_*b*_ in SEEG data and for each parcel pair *p*_*a*_, *p*_*b*_ in MEG data. Frequency pairs were chosen so that the ratio of their center frequencies lay within 5% deviation of the desired integer 1:*m* ratio.

CFS was computed as

$${PLV}_{CFS,a,b,m}=\frac{1}{N}|\sum_{t}exp[i∙(m∙\theta_{a,LF}-\theta_{b,HF})]|,$$ (3)

where *θ*_*a*,*LF*_ and *θ*_*b*,*HF*_ are the phases of the time series of contact/parcels. *θ*_*a*,*LF*_ was upsampled to match the sampling rate of the HF signal and then “phase-accelerated” by multiplication with *m* [39, 41]. Local CFS (*CFS*_*loc*_) was obtained where *a* = *b* and interareal CFS where *a* ≠ *b*.

The strength of PAC was quantified as

$${PLV}_{PAC,a,b}=\frac{1}{N}|\sum_{t}exp[i∙(\theta_{a,LF}-\theta_{b,HF,LF}^{env})]|,$$ (4)

where $\theta_{b,HF,LF}^{env}$ is the phase of the amplitude envelope of the HF signal filtered with a Morlet filter at LF and downsampled to match the LF signal’s sampling rate. Local PAC was obtained where *a* = *b*, interareal PAC where *a* ≠ *b*.

For both CFS and PAC, we obtained, for each subject and each frequency pair, surrogate values for each contact pair or parcel pair by rotating *θ_b,HF_* or $\theta_{b,HF,LF}^{env}$ analogously to what was done for PS and then calculated the means (PLV_CFS,surr_mean_, PLV_PAC,surr_mean_). As for PS, connections with a ratio *PLV*_*meas*_/*PLV*_*surr*_ of 2.42 or higher were identified as significant at α level 0.01, and connection density *K* was estimated as fraction of significant over possible connections, also as for PS.

### Analysis of amplitude–amplitude coupling

As a prerequisite to the removal of potentially spurious PAC (see next section), we estimated AC with PLV as the pairwise PS of LF-modulated amplitude envelopes of the HF signals. In order to do so, we obtained the LF-filtered amplitude envelopes of the HF signals $\theta_{i,HF,LF}^{env}$ and $\theta_{j,HF,LF}^{env}$, as described in the previous section, for all contact pairs or parcel pairs *i* ≠ *j* and all frequency pairs LF, HF and then computed the PS between these envelope time series using PLV. Significance for each connection and subject-level *K* were determined in the same manner as for PS.

### Removal of potentially spurious CFC connections

The core tenet of CFC is that it indicates an interaction between 2 distinct neuronal processes, and hence, CFC is genuine when there is evidence for the presence of 2 separate signals. Conversely, CFC may be spurious if evidence of the presence of 2 separate signals is not confirmed, and it remains a possibility that an observation of interareal CFC between 2 signals is due to at least one of them being nonsinusoidal and “leaking” via PS or AC to the other one. We developed a connection-by-connection test for whether interareal CFC can unambiguously be attributed to 2 separable signals. In this test, observations of interareal CFC are discarded between any 2 such signals that are also connected by both interareal 1:1 PS/AC and local CFC at one or both of the locations in a “triangle motif” (see Fig 1D–1H). Since such a test also may remove “ambiguous” cases, in which interareal CFC is genuine but nevertheless part of a triangle motif (see Fig 1G), our test is conservative in the sense that it minimizes false positives while possibly leading to false negatives and thus provides a lower-bound estimate for the number of genuine connections.

Interareal CFS was removed when there was a triangle motif of (significant) local CFS at the LF location and (significant) interareal HF PS or when there was local CFS at the HF location and significant interareal LF PS (see Fig 1E–1H). Likewise, interareal PAC was removed when there was a triangle motif of either local PAC at the HF location and LF interareal PS or when there was local PAC at the LF location and interareal HF AC.

### Single-subject analysis of CFC

In MEG data, we first identified a parcel pair *p*_*1*_, *p*_*2*_ with strong 1:2 α:β CFS across subjects. Next, we selected a representative subject and filtered that subject’s broadband (BB) time series with Morlet filters (*m* = 5) in *p*_1_ at LF = 11 Hz and in *p*_2_ at HF = 22 Hz to obtain NB time series. We then identified the largest α oscillation peaks in the real-valued LF time series and averaged time-locked data segments of 1,000-ms length centered around these α peaks (*N* = 62) in order to identify LF-peak–locked oscillations without a contribution from filtering artifacts.

The frequency content of the averaged BB time series at *p*_1_ and *p*_2_ was then visualized in TF plots obtained by Morlet-filtering (*m* = 5) the averaged BB time series at frequencies from 4 to 48 Hz (in steps of 1 Hz) and taking the amplitude *A* of the filtered time series. From this, we calculated, in the baseline windows from −500 ms to −200 ms and 200 ms to 500 ms relative to the peak, the mean and SD *A*_*BL_mean*_ and *A*_*BL_SD*_ and computed the z-score for *A* at each of the 1,000 time points:

$$z=\frac{A-A_{BL\_mean}}{A_{SD\_mean}}.$$

To corroborate the TF-analysis–based inferences of CFS, we also estimated the conventional 1:2 CFS (see Methods, Analysis of local and interareal of CFC: PAC and CFS) with PLV between the NB time series of *p*_1_ and *p*_2_ in sample-by-sample sliding time windows of 200 ms length, i.e., with phase differences concatenated across samples within the time windows and across the LF-peak–locked data segments. To estimate the null-hypothesis CFS and its distribution, the above analysis was performed with randomly picked data segments of equal numbers of samples. As described in Methods, Analysis of interareal phase synchronization, CFS was deemed significant when PLV_meas_ > 2.42 × PLV_surr_mean_.

In SEEG data, we identified a pair of electrode contacts *c*_1_, *c*_2_ (from different electrode shafts) with strong 1:5 α:γ PAC in a single subject. We filtered that subject’s BB times series in *c*_1_ at LF = 11 Hz and in *c*_2_ at HF = 55 Hz to obtain LF (α) and HF (γ) NB time series and in *c*_1_ identified the largest α troughs (*N* = 108). Centered around these troughs, we again averaged time-locked data segments of 1,000-ms length and obtained a TF plot of the average of time-locked BB time series in *c*_1_. From both contacts’ BB time series, we further constructed BB amplitude-average TF plots by filtering the BB time series at frequencies from 20 to 200 Hz and for each of these frequencies averaging the amplitude over segments time-locked to the LF (α) trough.

To again corroborate the TF- analysis–based inferences of PAC, we estimated 1:5 PAC conventionally between the LF NB time series in *c*_1_ and the averaged LF-filtered amplitude envelope of HF NB time series in *c*_2_ (see Methods, Analysis of local and interareal of CFC: PAC and CFS) in 200-ms sliding windows, concatenated across segments as for CFS. As for CFS, we estimated the null hypothesis and deemed PAC significant where PLV_meas_ > 2.42 × PLV_surr_mean_.

### Group-level statistics

To represent these data at the group level, we averaged the individual PLV and *K* values obtained as described above to obtain group-level *GS* and connection density *K*. In SEEG, these values were weighted with the number of contacts for each subject, which is equivalent to pooling all contacts across subjects. For estimating the group-level statistics of coupling strength and *K* for PS, AC, CFS, PAC, CFS_local_, and PAC_local_, group-level upper and lower confidence limits (2.5% and 97.5%) were computed with a bootstrapping approach using *N* = 1,000 resamplings, with replacement of the subjects in the cohort separately for each LF and for each frequency ratio LF:HF, again weighting values from SEEG with the number of contacts.

Since it can be expected that in the absence of any genuine interactions, 1% of observed edges would be false positives at the significance level *p* < 0.01, we subtracted 1% from the connection density *K* of significant connections for all reported and visualized *K* values. Corrected *K* values were computed as the fraction of the remaining significant connections divided by number of possible connections after removing all putatively spurious connections using the approach described in Methods, Removal of potentially spurious CFC connections.

### Computation of CFC in distance bins

For both SEEG and MEG data, we divided all channel/parcel pairs into 3 distance bins of equal numbers of connections using Euclidian distance between channel or parcel pairs. For SEEG data, the distance bins were 2–4 cm, 4–5.6 cm, and 5.6–13.7 cm, and for MEG data, these were 0–6.3 cm, 6.3–9.1cm, and 9.1–17.7 cm. We then computed the GS and *K* values separately in these distance bins as described above. We tested CFS and PAC for significant differences of *K* between all 3 pairs of distance bins with a Wilcoxon signed-rank test (*p* < 0.05, corrected for multiple comparisons across frequencies and combinations with Benjamini–Hochberg).

### Estimation of CFC in distinct cortical layers in SEEG data

We divided SEEG electrode contacts to 2 laminar depths based on the Grey Matter Proximity Index (GMPI), which is the distance between contact position and the nearest vertex of the white-gray surface, normalized by the cortical thickness in that point [91]. GMPI = 1 thus indicates the pial surface, and GMPI = 0 indicates the surface between gray and white matter. Based on this depth along the cortical gray matter, contacts within 0.5 < GMPI < 1.2 were marked as “superficial” and those within −0.3 < GMPI < 0 as “deep.” We analyzed interareal CFC among electrode pairs in 4 groups: 1) in which both electrode contacts were superficial, 2) in which both contacts were deep, 3) in which the LF contact was superficial and the HF contact deep, and 4) in which the LF contact was deep and the HF superficial. We tested CFS and PAC for significant difference of *K* values between all 6 pairs of laminar depth combinations with a Wilcoxon signed-rank test (*p* < 0.05, corrected for multiple comparisons across frequencies and combinations with Benjamini–Hochberg).

### Correlation of CFC in MEG with laminar depth in SEEG data

In order to elucidate the cortical sources contributing to the CFC connectomes observed with MEG, we performed an analysis to assess the correlation of these connectomes with those in layer-specific SEEG connectomes. We here focused on the frequency pairs that showed the most robust observations of interareal CFS and PAC interactions in both SEEG and MEG, namely 1:2–1:3 CFS and 1:2–1:4 PAC for the LF peak in the α band. We estimated for each of the selected frequency pairs the degree for each parcel in MEG as the number of connections as a measure of its centrality in the CFC network [80] and averaged degree values over frequency pairs of the same ratio. For SEEG data, we again divided the CFC connectome into the same 4 laminar depth combinations based on the localization of LF and HF electrode contacts in deep or superficial layers as described in Estimation of CFC in distinct cortical layers in SEEG data and then estimated parcel degrees within these subconnectomes and averaged, for each ratio and in each group, the degree values over frequency pairs. We then estimated the correlation of degree values in MEG CFC connectomes with those in each of the 4 SEEG groups using a Spearman rank correlation test, correcting for multiple comparisons with Benjamini–Hochberg. Fisher z-transform was further used to test differences between correlation values within SEEG depth combinations of the same ratio.

### Estimation of functional organization of CFC networks

To investigate the functional organization of CFC networks, we identified the brain regions that served predominantly as either LF or HF hubs. We achieved this by using 2 complimentary approaches: “relative directed degree” and “parcel directionality.”

#### Estimation of relative directed degree

First, in order to be able to compare SEEG and MEG data, all electrode contacts in SEEG, and all parcels of the 200-parcel atlas that had been used so far in MEG were collapsed to the 148 parcels of the Destrieux atlas [73]. Then, for each LF–HF combination, we estimated for each parcel *p* the graph-theoretic measures “relative in-degree” and “relative out-degree” [80], indicating how often that parcel was either the HF or LF node, respectively, of a CFC connection. The relative in-degree or out-degree of a parcel was defined as the fraction of significant connections *N*_*C*_ for which that parcel was the HF node or LF node, respectively, over the total possible number of possible connections *N*_*C*,*pot*_. In SEEG data, *N*_*E*,*pot*,*S*_ was for each subject *S* the sum of possible, i.e., not excluded, connections from contacts assigned to that parcel to electrodes assigned to other parcels, and *N*_*C*,*pot*_ was obtained by adding possible CFS or PAC connections over all subjects. In MEG, *N*_*C*,*pot*_ was simply the number of possible, i.e., not excluded, connections of one parcel to other parcels times the number of subject datasets. Finally, the “relative directed degree” was computed as the difference between relative in-degree and out-degree. Positive values therefore indicated that a parcel was predominantly a HF hub, and a negative value indicated that it was predominantly a LF hub. Relative directed degree values were collapsed over frequency bands for visualization. In order to assess similarity between SEEG and MEG data and between CFS and PAC, we computed the correlation of relative directed degree values across parcels using Spearman test (*p* < 0.05).

#### Estimation of parcel directionality

In this approach, we first estimated, for each not-excluded pair of parcels *p*_1_, *p*_2_ of the Destrieux atlas, the LF–HF directionality *Dir*_*LH*_ as the difference in the strengths of CFC connections:

$${Dir}_{LH}\;(LF,HF,p_{1},p_{2})=\frac{1}{N}(\sum^{N}{PLV}_{CF}(LF,HF,p_{2},p_{1})-\sum^{N}{PLV}_{CF}(LF,HF,p_{1},p_{2})),$$ (5)

i.e., the total strength of connections in which *p*_1_ was the HF node and *p*_2_ the LF node minus the total strength of connections in which *p*_2_ was the HF node and *p*_1_ the LF node. *N* was the number of connections between the 2 parcels, equivalent to *N*_*C*,*pot*_ defined above. For all pairs (*p*_1_, *p*_2_) in SEEG for which *N*_*C*_ < 8, *Dir*_*LH*_ was set to 0.

We then tested, for all frequency pairs, all nonzero values of *Dir*_*LH*_ (*LF*, *HF*, *p*_1_, *p*_2_) for significance value using a permutation test. In each permutation (*N* = 1,000), the strengths of all connections *PLV*_*CF*_ (*LF*, *HF*, *p*_1_, *p*_2_) and *PLV*_*CF*_ (*LF*, *HF*, *p*_2_, *p*_1_) between 2 parcels *p*_1_, *p*_2_ were pooled and randomly assigned to 2 groups *G*_1_, *G*_2_. The permutated directionality *Dir*_*LH*_ value was then computed as

$${Dir}_{LH,perm}(LF,HF,p_{1},p_{2})=\frac{1}{M}(\sum^{M}{PLV}_{CF}(LF,HF,G_{1})-\sum^{M}{PLV}_{CF}(HF,LF,G_{2})).$$

If the genuine *Dir*_*LH*_ value was larger than *Dir*_*LH*, *perm*_ in 95% of permutations, the directionality of the connection was deemed significant. A significant value of *Dir*_*LH*_ > 0 thus indicated, that between *p*_1_ and *p*_2_, those connections in which parcel *p*_1_ was the HF node and *p*_2_ was the LF node were significantly stronger than those in which it was the other around.

The overall directionality ${Dir}_{LH}^{p}$ of a parcel *p* was computed as the number of connections with other parcels for which its *Dir*_*LH*_ value was positive minus the number of those for which it was negative, divided by the total number *N* of possible connections. Thus, similar to the directed relative degree, a positive or negative value of ${Dir}_{LH}^{p}$ indicated a parcel being a HF hub or LF hub, respectively, in interareal CFC. Analogously to relative directed degree, the directionality values were collapsed over frequency bands for visualization, and their similarity between SEEG and MEG and between CFS and PAC was estimated using Spearman test across parcels (*p* < 0.05).

### Neuropsychological assessment and correlation of CFC with neuropsychological test results

A subset of the participants of the MEG recordings performed a set of well-validated neuropsychological assessments (*N* = 16–18 for each test). Four tests were used from the Wechsler Adult Intelligence Scale–III (WAIS–III) battery [112]: the Digit Span Forward subtest evaluates verbal short-term memory, while the Digit Span Backward and Letter-Number Sequencing (LNS) subtests evaluate verbal WM, and the Digit Symbol–Coding subtest measures visual psychomotor processing speed. The TMT [113] is composed of 2 parts, A and B, of which the TMT-A measures visual scanning and processing speed while the TMT-B measures cognitive flexibility, visual scanning, and processing speed. The Zoo Map Test [114] measures the participant’s planning capability and speed. For the correlation analysis, the test scores were inverted for the TMT and Zoo Map tests, which measure processing speed, so that the higher values were indexing better performance as in the first 4 tests. To investigate the correlation of the CFC with psychophysical performance, we then computed the correlation of subject’s test scores with their individual GS using Spearman rank correlation test (*p* < 0.05). To consider the effect of multiple comparisons across all tests performed, we estimated how many of the total number of the observed significant findings were predicted to be observable by chance. In total, across the 8 neuropsychological tests and the CFC frequency pairs, the probability for finding the observed number of significant observations (170) by chance was *p* = 0.0051. Thus, we consider the CFS overall to be significantly correlated with neuropsychological tests. The asterisks in Fig 8 indicate the most significant observations (34) exceeding the number of significant observations (136) predicted to be observable by chance at *p* = 0.05. The total number of significant observations for PAC was 45, i.e., below the number expected by chance at *p* = 0.5. Thus, PAC, at least in such widespread testing, is overall not correlated with neuropsychological test performance.

## Supporting information

S1 Fig Workflow for SEEG data. Workflow for the processing and analysis of SEEG data, flowing from top to bottom. SEEG, stereoelectroencephalography.
(EPS)

S2 Fig Workflow for MEG data. Workflow for the processing and analysis of MEG data, flowing from top to bottom. MEG, magnetoencephalography
(EPS)

S3 Fig GS of interareal CFS and PAC. (a) The GS (mean strength of all connections of all subjects) of interareal CFC with 95% confidence limits for ratio 1:2 (top row) and ratios 1:3–1:7 (bottom row). (b) GS of interareal CFC at ratio 1:2 (top row) and 1:3 (bottom row) in 3 distance bins, as in Fig 4. (c) GS of interareal CFC at ratio 1:2 (top row) and 1:3 (bottom row) in SEEG among different groups of electrode pairs, based on their location in deep or superficial layers, as in Fig 6. Plot data and underlying connectome data are available online at https://datadryad.org/stash/dataset/doi:10.5061/dryad.0k86k80. CFC, cross-frequency coupling; CFS, cross-frequency phase synchrony; GS, graph strength; PAC, phase–amplitude coupling; SEEG, stereoelectroencephalography.
(EPS)

S4 Fig 1:1 PS in SEEG and MEG. (a) The mean *GS* of 1:1 PS (top) and *K* (bottom) in SEEG data with 95% confidence limits estimated with the wPLI for all connections (left) and separately in three distance bins (right). (b) Same in MEG data. (c–d) Phase synchrony in SEEG data and MEG data, respectively, estimated with the PLV. Plot data and underlying connectome data are available online at https://datadryad.org/stash/dataset/doi:10.5061/dryad.0k86k80. *GS*, graph strength; MEG, magnetoencephalography; PLV, phase-locking value; PS, phase synchrony; SEEG, stereoelectroencephalography; wPLI, weighted phase-lag index.
(EPS)

S5 Fig Local CFS and PAC in SEEG and MEG data. (a) The mean graph strength *(GS)* and fraction of significant parcels (*K)* with 95% confidence limits (shaded) of local CFS in SEEG data for coupling ratios 1:2 (top) and 1:3–1:7 (bottom). Values are shown with 95% confidence limits. (b) Same for local CFS in MEG data. (c–d) Same for local PAC in SEEG data and MEG data, respectively. (e–h) The same *K* values as shown in (a)–(d) plotted in matrices for the whole spectral connectome. Plot data and underlying connectome data are available online at https://datadryad.org/stash/dataset/doi:10.5061/dryad.0k86k80. CFS, cross-frequency PS; GS, graph strength; MEG, magnetoencephalography; PAC, phase–amplitude coupling; PS, phase synchrony; SEEG, stereoelectroencephalography.
(EPS)

S6 Fig ACs. (a) GS and *K* for ACs in SEEG data for ratios of 1:2 to 1:7. The same data are plotted as a function of the envelope frequency (top row) and the modulating frequency (bottom row). (b) Same for MEG data. Plot data and underlying connectome data are available online at https://datadryad.org/stash/dataset/doi:10.5061/dryad.0k86k80. AC, amplitude envelope correlation; GS, graph strength; MEG, magnetoencephalography; SEEG, stereoelectroencephalography.
(EPS)

S7 Fig Interareal CFS and PAC when PLV is used for removing spurious connections. (a) Connection density *K* of interareal CFS in SEEG data before (left) and after removing possible spurious connections (right) using the PLV as a metric for phase synchrony. (b) Same for interareal PAC in SEEG data. (c–d) Same for interareal CFS and PAC in MEG data. Plot data and underlying connectome data are available online at https://datadryad.org/stash/dataset/doi:10.5061/dryad.0k86k80. CFS, cross-frequency PS; MEG, magnetoencephalography; PAC, phase–amplitude coupling; PLV, phase-locking value; SEEG, stereoelectroencephalography.
(EPS)

S8 Fig CFS in eyes-open and eyes-closed RS. (a) The mean *n*:*m* CFS *GS* (left), *K* before correction (middle), and *K* after correction (right, using PLV as PS metric) of interareal 1:2–1:4 CFS in eyes-open RS MEG data. (b) The same in eyes-closed RS MEG data. Plot data and underlying connectome data are available online at https://datadryad.org/stash/dataset/doi:10.5061/dryad.0k86k80. CFS, cross-frequency PS; GS, graph strength; MEG, magnetoencephalography; PLV, phase-locking value; PS, phase synchrony; RS, resting state.
(EPS)

S9 Fig Correlations between MEG data and laminar SEEG data. Correlation of parcel degree values of MEG CFC connectomes with parcel degree values of SEEG CFC connectomes connecting electrodes in either both in more superficial layers, both in deeper layers, from superficial to deep or deep to superficial layers (Spearman rank correlation test, ***p < 0.001, **p < 0.01, *p < 0.05, (*)*p* < 0.05, n.s. after correction with Benjamini–Hochberg). The black bar denotes when 2 correlation values for the same ratio were found to be significantly different by a Fisher z-transform test (*z* > 1.96). Plot data and underlying connectome data are available online at https://datadryad.org/stash/dataset/doi:10.5061/dryad.0k86k80. CFC, cross-frequency coupling; MEG, magnetoencephalography; n.s., nonsignificant; SEEG, stereoelectroencephalography.
(EPS)

S10 Fig Asymmetric directional connectivity in CFC networks. The organization of CFC networks as measured with directionality between LF and HF hubs. Averaged LF versus HF directionality values for each parcel. The values indicate whether parcel is a directional hub for LF (blue) or for HF (red) in interareal CFC networks. Top row: directionality for CFS and PAC networks at ratio 1:2 connecting θ:α and α:β frequencies. Bottom row: directionality for CFS and PAC at ratio 1:3, connecting δ:α and α:γ frequencies. Directional connections of CFS and PAC networks reflect their structure in brain anatomy and show similar opposite directional connections connecting anterior and posterior brain regions, as seen in degree hub analysis (see Fig 7). Plot data and underlying connectome data are available online at https://datadryad.org/stash/dataset/doi:10.5061/dryad.0k86k80. CFC, cross-frequency coupling; CFS, cross-frequency PS; HF, high frequency; LF, low frequency; PAC, phase–amplitude coupling.
(EPS)

S11 Fig Correlation of PAC with neuropsychological test scores. The correlation or PAC strength in MEG data with neuropsychological test scores (Spearman rank correlation test, *p* < 0.05). Red color indicates a positive correlation, while blue indicates a negative correlation, as in Fig 8. Correlations that do not reach statistical significance are masked with lower opacity. No correlations were significant after correcting for multiple comparisons with Benjamini–Hochberg. Plot data and underlying connectome data and neuropsychological data are available online at https://datadryad.org/stash/dataset/doi:10.5061/dryad.0k86k80. MEG, magnetoencephalography; PAC, phase–amplitude coupling.
(EPS)

## Abbreviations

AC: amplitude envelope correlation
CFC: cross-frequency coupling
CFS: cross-frequency PS
CS: central sulcus
EEG: electroencephalograpy
fMRI: functional magnetic resonance imaging
FPR: false positive rate
GS: graph strength
HF: high frequency
iTS: inferior TS
LF: low frequency
LFP: local field potential
lPFC: lateral prefrontal cortex
MEG: magnetoencephalography
MPC: medial parietal cortex
mTG: middle temporal gyrus
NB: narrowband
PAC: phase–amplitude coupling
PLV: phase-locking value
PPC: posterior parietal cortex
PS: phase synchrony
RS: resting state
RSN: RS network
SEEG: stereoelectroencephalography
SM: somatomotor
supTS: superior TS
TF: time frequency
TFR: TF representation
TMT: Trail-Making Test
TS: temporal sulcus
vmCS: ventromedial CS
WM: working memory
wPLI: weighted phase-lag index

## References

[1] Singer W. Neuronal synchrony: A versatile code for the definition of relations? Neuron. 1999;24(1): 49–65, 111–25. 10.1016/s0896-6273(00)80821-1 10677026

[2] Fries P. Rhythms for cognition: Communication through coherence. Neuron. 2015;88(1): 220–235. 10.1016/j.neuron.2015.09.034 26447583 PMC4605134

[3] Brookes MJ, Woolrich M, Luckhoo H, Price D, Hale JR, et al Investigating the electrophysiological basis of resting state networks using magnetoencephalography. Proc Natl Acad Sci U S A. 2011;108(40): 16783–16788. 10.1073/pnas.1112685108 21930901 PMC3189080

[4] Hipp JF, Hawellek DJ, Corbetta M, Siegel M, Engel AK. Large-scale cortical correlation structure of spontaneous oscillatory activity. Nat Neurosci. 2012;15(6): 884–890. 10.1038/nn.3101 22561454 PMC3861400

[5] de Pasquale F, Della Penna S, Snyder AZ, Lewis C, Mantini D, et al Temporal dynamics of spontaneous MEG activity in brain networks. Proc Natl Acad Sci U S A. 2010;107(13): 6040–6045. 10.1073/pnas.0913863107 20304792 PMC2851876

[6] Arnulfo G, Hirvonen J, Nobili L, Palva S, Palva JM. Phase and amplitude correlations in resting-state activity in human stereotactical EEG recordings. Neuroimage. 2015;112: 114–127. 10.1016/j.neuroimage.2015.02.031 25721426

[7] Siems M, Pape A, Hipp JF, Siegel M. Measuring the cortical correlation structure of spontaneous oscillatory activity with EEG and MEG. Neuroimage. 2016;129: 345–355. 10.1016/j.neuroimage.2016.01.055 26827813

[8] de Pasquale F, Della Penna S, Sporns O, Romani GL, Corbetta M. A dynamic core network and global efficiency in the resting human brain. Cereb Cortex. 2016;26(10): 4015–33. 10.1093/cercor/bhv185 26347485 PMC5027996

[9] Fell J, Axmacher N. The role of phase synchronization in memory processes. Nat Rev Neurosci. 2011;12(2): 105–118. 10.1038/nrn2979 21248789

[10] Palva S, Palva JM. Discovering oscillatory interaction networks with M/EEG: Challenges and breakthroughs. Trends Cogn Sci. 2012;16(4): 219–230. 10.1016/j.tics.2012.02.004 22440830

[11] Siegel M, Donner TH, Engel AK. Spectral fingerprints of large-scale neuronal interactions. Nat Rev Neurosci. 2012;13(2): 121–134. 10.1038/nrn3137 22233726

[12] Harris AZ, Gordon JA. Long-range neural synchrony in behavior. Annu Rev Neurosci. 2015;38: 171–194. 10.1146/annurev-neuro-071714-034111 25897876 PMC4497851

[13] Sadaghiani S, Kleinschmidt A. Brain networks and α-oscillations: Structural and functional foundations of cognitive control. Trends Cogn Sci (Regul Ed). 2016;20(11): 805–817. 10.1016/j.tics.2016.09.004 27707588

[14] Palva S, Palva JM. New vistas for alpha-frequency band oscillations. Trends Neurosci. 2007;30(4): 150–158. 10.1016/j.tins.2007.02.001 17307258

[15] Bastos AM, Vezoli J, Bosman CA, Schoffelen JM, Oostenveld R, et al Visual areas exert feedforward and feedback influences through distinct frequency channels. Neuron. 2015;85(2): 390–401. 10.1016/j.neuron.2014.12.018 25556836

[16] Jensen O, Bonnefond M, Marshall TR, Tiesinga P. Oscillatory mechanisms of feedforward and feedback visual processing. Trends Neurosci. 2015;38(4): 192–194. 10.1016/j.tins.2015.02.006 25765320

[17] Palva S, Palva JM. Functional roles of alpha-band phase synchronization in local and large-scale cortical networks. Front Psychol. 2011;2: 204 10.3389/fpsyg.2011.00204 21922012 PMC3166799

[18] Klimesch W. Alpha-band oscillations, attention, and controlled access to stored information. Trends Cogn Sci. 2012;16(12): 606–617. 10.1016/j.tics.2012.10.007 23141428 PMC3507158

[19] Womelsdorf T, Everling S. Long-range attention networks: Circuit motifs underlying endogenously controlled stimulus selection. Trends Neurosci. 2015;38(11): 682–700. 10.1016/j.tins.2015.08.009 26549883

[20] Vidal JR, Chaumon M, O'Regan JK, Tallon-Baudry C. Visual grouping and the focusing of attention induce gamma-band oscillations at different frequencies in human magnetoencephalogram signals. J Cogn Neurosci. 2006;18(11): 1850–1862. 10.1162/jocn.2006.18.11.1850 17069476

[21] Honkanen R, Rouhinen S, Wang SH, Palva JM, Palva S. Gamma oscillations underlie the maintenance of feature-specific information and the contents of visual working memory. Cereb Cortex. 2015;25(10): 3788–3801. 10.1093/cercor/bhu263 25405942

[22] Michalareas G, Vezoli J, van Pelt S, Schoffelen J, Kennedy H, et al Alpha-beta and gamma rhythms subserve feedback and feedforward influences among human visual cortical areas. Neuron. 2016;89(2): 384–397. 10.1016/j.neuron.2015.12.018 26777277 PMC4871751

[23] Palmer C, Zapparoli L, Kilner JM. A new framework to explain sensorimotor beta oscillations. Trends Cogn Sci (Regul Ed). 2016;20(5): 321–323. 10.1016/j.tics.2016.03.007 27026481 PMC5345740

[24] Abbasi O, Gross J. Beta-band oscillations play an essential role in motor–auditory interactions. Hum Brain Mapp. 2020;41: 656–665. Epub 22 10 2019 10.1002/hbm.24830 31639252 PMC7268072

[25] Deco G, Cabral J, Woolrich MW, Stevner ABA, van Hartevelt TJ, et al Single or multiple frequency generators in on-going brain activity: A mechanistic whole-brain model of empirical MEG data. Neuroimage. 2017;152: 538–550. 10.1016/j.neuroimage.2017.03.023 28315461 PMC5440176

[26] Palva S, Palva JM. Roles of brain criticality and multiscale oscillations in sensorimotor predictions. Trends Neurosci. 2018;41(10): 729–743. 10.1016/j.tins.2018.08.008 30274607

[27] Hahn G, Ponce-Alvarez A, Deco G, Aertsen A, Kumar A. Portraits of communication in neuronal networks. Nature Reviews Neuroscience. 2019;20(2): 117–127. 10.1038/s41583-018-0094-0 30552403

[28] Fox MD, Snyder AZ, Vincent JL, Corbetta M, Van Essen DC, et al The human brain is intrinsically organized into dynamic, anticorrelated functional networks. Proc Natl Acad Sci U S A. 2005;102(27): 9673–9678. 10.1073/pnas.0504136102 15976020 PMC1157105

[29] Raichle ME. The brain's default mode network. Annu Rev Neurosci. 2015;38(1): 433–447. 10.1146/annurev-neuro-071013-014030 25938726

[30] Hipp JF, Siegel M. BOLD fMRI correlation reflects frequency-specific neuronal correlation. Curr Biol. 2015;25(10): 1368–1374. 10.1016/j.cub.2015.03.049 25936551

[31] Zhigalov A, Arnulfo G, Nobili L, Palva S, Palva JM. Modular co-organization of functional connectivity and scale-free dynamics in the human brain. Network Neuroscience. 2017;1(2): 143–165. 10.1162/NETN_a_00008 29911674 PMC5988393

[32] Betti V, Corbetta M, de Pasquale F, Wens V, Della Penna S. Topology of functional connectivity and hub dynamics in the beta band as temporal prior for natural vision in the human brain. Journal of Neuroscience. 2018;38(15): 3858–3871. 10.1523/JNEUROSCI.1089-17.2018 29555851 PMC6705907

[33] Jensen O, Colgin LL. Cross-frequency coupling between neuronal oscillations. Trends in Cognitive Sciences. 2007;11(7): 267–269. 10.1016/j.tics.2007.05.003 17548233

[34] Schroeder CE, Lakatos P. Low-frequency neuronal oscillations as instruments of sensory selection. Trends Neurosci. 2009;32(1): 9–18. 10.1016/j.tins.2008.09.012 19012975 PMC2990947

[35] Canolty RT, Knight RT. The functional role of cross-frequency coupling. Trends Cogn Sci. 2010;14(11): 506–515. 10.1016/j.tics.2010.09.001 20932795 PMC3359652

[36] Lisman JE, Jensen O. The theta-gamma neural code. Neuron. 2013;77(6): 1002–1016. 10.1016/j.neuron.2013.03.007 23522038 PMC3648857

[37] Hyafil A, Giraud AL, Fontolan L, Gutkin B. Neural cross-frequency coupling: Connecting architectures, mechanisms, and functions. Trends Neurosci. 2015;38(11): 725–740. 10.1016/j.tins.2015.09.001 26549886

[38] Tass P, Rosenblum MG, Weule J, Kurths J, Pikovsky A, et al Detection of n:M phase locking from noisy data: Application to magnetoencephalography. Phys Rev Lett. 1998;81(15): 3291–94. 10.1103/PhysRevLett.81.3291

[39] Palva JM, Palva S, Kaila K. Phase synchrony among neuronal oscillations in the human cortex. J Neurosci. 2005;25(15): 3962–3972. 10.1523/JNEUROSCI.4250-04.2005 15829648 PMC6724920

[40] Sauseng P, Klimesch W, Gruber WR, Birbaumer N. Cross-frequency phase synchronization: A brain mechanism of memory matching and attention. Neuroimage. 2008;40(1): 308–317. 10.1016/j.neuroimage.2007.11.032 18178105

[41] Siebenhühner F, Wang SH, Palva JM, Palva S. Cross-frequency synchronization connects networks of fast and slow oscillations during visual working memory maintenance. Elife. 2016;5: e13451 10.7554/eLife.13451 27669146 PMC5070951

[42] Roux F, Uhlhaas PJ. Working memory and neural oscillations: Alpha-gamma versus theta-gamma codes for distinct WM information? Trends Cogn Sci. 2014;18(1): 16–25. 10.1016/j.tics.2013.10.010 24268290

[43] Tort AB, Kramer MA, Thorn C, Gibson DJ, Kubota Y, et al Dynamic cross-frequency couplings of local field potential oscillations in rat striatum and hippocampus during performance of a T-maze task. Proc Natl Acad Sci U S A. 2008;105(51): 20517–20522. 10.1073/pnas.0810524105 19074268 PMC2629291

[44] Tort AB, Komorowski R, Eichenbaum H, Kopell N. Measuring phase-amplitude coupling between neuronal oscillations of different frequencies. J Neurophysiol. 2010;104(2): 1195–1210. 10.1152/jn.00106.2010 20463205 PMC2941206

[45] Belluscio MA, Mizuseki K, Schmidt R, Kempter R, Buzsaki G. Cross-frequency phase-phase coupling between theta and gamma oscillations in the hippocampus. J Neurosci. 2012;32(2): 423–435. 10.1523/JNEUROSCI.4122-11.2012 22238079 PMC3293373

[46] Scheffer-Teixeira R, Belchior H, Caixeta F, Souza BC, Ribeiro S, et al Theta phase modulates multiple layer-specific oscillations in the CA1 region. Cerebral Cortex. 2012;22(10): 2404–2414. 10.1093/cercor/bhr319 22079925

[47] Scheffer-Teixeira R, Tort ABL. Unveiling fast field oscillations through comodulation. ENeuro. 2017;4(4): eneuro.0079–17.2017. 10.1523/eneuro.0079-17.2017 28785730 PMC5545523

[48] Canolty RT, Edwards E, Dalal SS, Soltani M, Nagarajan SS, et al High gamma power is phase-locked to theta oscillations in human neocortex. Science. 2006;313(5793): 1626–1628. 10.1126/science.1128115 16973878 PMC2628289

[49] Axmacher N, Henseler MM, Jensen O, Weinreich I, Elger CE, et al Cross-frequency coupling supports multi-item working memory in the human hippocampus. Proc Natl Acad Sci U S A. 2010;107(7): 3228–3233. 10.1073/pnas.0911531107 20133762 PMC2840289

[50] Watrous AJ, Deuker L, Fell J, Axmacher N. Phase-amplitude coupling supports phase coding in human ECoG. Elife. 2015;4: eLife.07886 10.7554/eLife.07886 26308582 PMC4579288

[51] Bahramisharif A, Jensen O, Jacobs J, Lisman J. Serial representation of items during working memory maintenance at letter-selective cortical sites. PLoS Biol. 2018;16(8): e2003805 10.1371/journal.pbio.2003805 30110320 PMC6093599

[52] Roux F, Wibral M, Singer W, Aru J, Uhlhaas PJ. The phase of thalamic alpha activity modulates cortical gamma-band activity: Evidence from resting-state MEG recordings. J Neurosci. 2013;33(45): 17827 10.1523/JNEUROSCI.5778-12.2013 24198372 PMC3818555

[53] Florin E, Baillet S. The brain's resting-state activity is shaped by synchronized cross-frequency coupling of neural oscillations. Neuroimage. 2015;111: 26–35. 10.1016/j.neuroimage.2015.01.054 25680519 PMC4387013

[54] Park H, Ince RA, Schyns PG, Thut G, Gross J. Frontal top-down signals increase coupling of auditory low-frequency oscillations to continuous speech in human listeners. Curr Biol. 2015;25(12): 1649–1653. 10.1016/j.cub.2015.04.049 26028433 PMC4503802

[55] Park H, Lee DS, Kang E, Kang H, Hahm J, et al Formation of visual memories controlled by gamma power phase-locked to alpha oscillations. Sci Rep. 2016;6: 28092 10.1038/srep28092 27306959 PMC4910116

[56] Keitel C, Thut G, Gross J. Visual cortex responses reflect temporal structure of continuous quasi-rhythmic sensory stimulation. Neuroimage. 2017;146: 58–70. 10.1016/j.neuroimage.2016.11.043 27867090 PMC5312821

[57] Palva JM, Palva S. Functional integration across oscillation frequencies by cross‐frequency phase synchronization. Eur J Neurosci. 2017;48(7): 2399–2406. 10.1111/ejn.13767 29094462

[58] Nikulin VV, Brismar T. Phase synchronization between alpha and beta oscillations in the human electroencephalogram. Neuroscience. 2006;137(2): 647–657. 10.1016/j.neuroscience.2005.10.031 16338092

[59] Sauseng P, Klimesch W, Heise KF, Gruber WR, Holz E, et al Brain oscillatory substrates of visual short-term memory capacity. Curr Biol. 2009;19(21): 1846–1852. 10.1016/j.cub.2009.08.062 19913428

[60] van der Meij R, Kahana M, Maris E. Phase-amplitude coupling in human electrocorticography is spatially distributed and phase diverse. J Neurosci. 2012;32(1): 111–123. 10.1523/JNEUROSCI.4816-11.2012 22219274 PMC6621324

[61] Kramer MA, Tort ABL, Kopell NJ. Sharp edge artifacts and spurious coupling in EEG frequency comodulation measures. J Neurosci Methods. 2008;170(2): 352–357. 10.1016/j.jneumeth.2008.01.020 18328571

[62] Aru J, Aru J, Priesemann V, Wibral M, Lana L, et al Untangling cross-frequency coupling in neuroscience. Curr Opin Neurobiol. 2015;31: 51–61. 10.1016/j.conb.2014.08.002 25212583

[63] van Driel J, Cox R, Cohen MX. Phase-clustering bias in phase–amplitude cross-frequency coupling and its removal. Journal of Neuroscience Methods. 2015;254: 60–72. 10.1016/j.jneumeth.2015.07.014 26231622

[64] Gerber EM, Sadeh B, Ward A, Knight RT, Deouell LY. Non-sinusoidal activity can produce cross-frequency coupling in cortical signals in the absence of functional interaction between neural sources. PLoS ONE. 2016;11(12): e0167351 10.1371/journal.pone.0167351 27941990 PMC5152905

[65] Scheffer-Teixeira R, Tort AB. On cross-frequency phase-phase coupling between theta and gamma oscillations in the hippocampus. Elife. 2016;5: 10.7554/eLife.20515. 10.7554/eLife.20515 27925581 PMC5199196

[66] Lozano-Soldevilla D, Ter Huurne N, Oostenveld R. Neuronal oscillations with non-sinusoidal morphology produce spurious phase-to-amplitude coupling and directionality. Front Comput Neurosci. 2016;10: 87 10.3389/fncom.2016.00087 27597822 PMC4992698

[67] Cole SR, Voytek B. Brain oscillations and the importance of waveform shape. Trends Cogn Sci. 2017;21(2): 137–149. 10.1016/j.tics.2016.12.008 28063662

[68] Jensen O, Spaak E, Park H. Discriminating valid from spurious indices of phase-amplitude coupling. ENeuro. 2017;3(6): 10.1523/eneuro.0334. 10.1523/eneuro.0334-16.2016 28101528 PMC5237829

[69] Nikulin VV, Linkenkaer-Hansen K, Nolte G, Lemm S, Muller KR, et al A novel mechanism for evoked responses in the human brain. Eur J Neurosci. 2007;25(10): 3146–3154. 10.1111/j.1460-9568.2007.05553.x 17561828

[70] Cole S, Voytek B. Cycle-by-cycle analysis of neural oscillations. J Neurophysiol. 2019;122(2): 849–861. 10.1152/jn.00273.2019 31268801

[71] Petkoski S, Palva JM, Jirsa VK. Phase-lags in large scale brain synchronization: Methodological considerations and in-silico analysis. PLoS Comput Biol. 2018;14(7): e1006160 10.1371/journal.pcbi.1006160 29990339 PMC6039010

[72] Palva JM, Monto S, Kulashekhar S, Palva S. Neuronal synchrony reveals working memory networks and predicts individual memory capacity. Proc Natl Acad Sci U S A. 2010;107(16): 7580–7585. 10.1073/pnas.0913113107 20368447 PMC2867688

[73] Destrieux C, Fischl B, Dale A, Halgren E. Automatic parcellation of human cortical gyri and sulci using standard anatomical nomenclature. Neuroimage. 2010;53(1): 1–15. 10.1016/j.neuroimage.2010.06.010 20547229 PMC2937159

[74] Muthukumaraswamy SD, Singh KD. Visual gamma oscillations: The effects of stimulus type, visual field coverage and stimulus motion on MEG and EEG recordings. Neuroimage. 2013;69: 223–230. 10.1016/j.neuroimage.2012.12.038 23274186

[75] Vinck M, Oostenveld R, van Wingerden M, Battaglia F, Pennartz CM. An improved index of phase-synchronization for electrophysiological data in the presence of volume-conduction, noise and sample-size bias. Neuroimage. 2011;55(4): 1548–1565. 10.1016/j.neuroimage.2011.01.055 21276857

[76] Palva JM, Wang SH, Palva S, Zhigalov A, Monto S, et al Ghost interactions in MEG/EEG source space: A note of caution on inter-areal coupling measures. NeuroImage. 2018;173: 632–643. 10.1016/j.neuroimage.2018.02.032 29477441

[77] Niedermeyer E, Lopes da Silva FH, editors. Electroencephalography: Basic principles, clinical applications, and related fields. Philadelphia, USA: Lippincott Williams & Wilkins; 2005.

[78] Hirvonen J, Wibral M, Palva JM, Singer W, Uhlhaas P, et al Whole-brain source-reconstructed MEG-data reveal reduced long-range synchronization in chronic schizophrenia. ENeuro. 2017;4(5): 0338 10.1523/eneuro.0338-17.2017 29085902 PMC5659261

[79] Hamalainen MS, Sarvas J. Realistic conductivity geometry model of the human head for interpretation of neuromagnetic data. IEEE Trans Biomed Eng. 1989;36(2): 165–171. 10.1109/10.16463 2917762

[80] Bassett DS, Bullmore E. Small-world brain networks. Neuroscientist. 2006;12(6): 512–523. 10.1177/1073858406293182 17079517

[81] Donnelly-Kehoe P, Saenger VM, Lisofsky N, Kühn S, Kringelbach ML, et al Reliable local dynamics in the brain across sessions are revealed by whole-brain modeling of resting state activity. Hum Brain Mapp. 2019;40(10): 2967–2980. 10.1002/hbm.24572 30882961 PMC6865451

[82] Lakatos P, Shah AS, Knuth KH, Ulbert I, Karmos G, et al An oscillatory hierarchy controlling neuronal excitability and stimulus processing in the auditory cortex. J Neurophysiol. 2005;94(3): 1904–1911. 10.1152/jn.00263.2005 15901760

[83] Lakatos P, Karmos G, Mehta AD, Ulbert I, Schroeder CE. Entrainment of neuronal oscillations as a mechanism of attentional selection. Science. 2008;320(5872): 110–113. 10.1126/science.1154735 18388295

[84] Vanhatalo S, Palva JM, Holmes MD, Miller JW, Voipio J, et al Infraslow oscillations modulate excitability and interictal epileptic activity in the human cortex during sleep. Proc Natl Acad Sci U S A. 2004;101(14): 5053–5057. 10.1073/pnas.0305375101 15044698 PMC387372

[85] Luo H, Liu Z, Poeppel D. Auditory cortex tracks both auditory and visual stimulus dynamics using low-frequency neuronal phase modulation. PLoS Biol. 2010;8(8): e1000445 10.1371/journal.pbio.1000445 20711473 PMC2919416

[86] Cohen MX, Elger CE, Fell J. Oscillatory activity and phase-amplitude coupling in the human medial frontal cortex during decision making. J Cogn Neurosci. 2009;21(2): 390–402. 10.1162/jocn.2008.21020 18510444

[87] Bollimunta A, Mo J, Schroeder CE, Ding M. Neuronal mechanisms and attentional modulation of corticothalamic alpha oscillations. J Neurosci. 2011;31(13): 4935–4943. 10.1523/JNEUROSCI.5580-10.2011 21451032 PMC3505610

[88] Buffalo EA, Fries P, Landman R, Buschman TJ, Desimone R. Laminar differences in gamma and alpha coherence in the ventral stream. Proc Natl Acad Sci U S A. 2011;108(27): 11262–11267. 10.1073/pnas.1011284108 21690410 PMC3131344

[89] Haegens S, Barczak A, Musacchia G, Lipton ML, Mehta AD, et al Laminar profile and physiology of the alpha rhythm in primary visual, auditory, and somatosensory regions of neocortex. J Neurosci. 2015;35(42): 14341–14352. 10.1523/JNEUROSCI.0600-15.2015 26490871 PMC4683691

[90] Senzai Y, Fernandez-Ruiz A, Buzsáki G. Layer-specific physiological features and interlaminar interactions in the primary visual cortex of the mouse. Neuron. 2019;101(3): 500–513.e5 10.1016/j.neuron.2018.12.009 30635232 PMC6367010

[91] Arnulfo G, Narizzano M, Cardinale F, Fato MM, Palva JM. Automatic segmentation of deep intracerebral electrodes in computed tomography scans. BMC Bioinformatics. 2015;16(1): 99-015-0511–6. 10.1186/s12859-015-0511-6 25887573 PMC4393625

[92] Hari R, Salmelin R. Magnetoencephalography: From SQUIDs to neuroscience: Neuroimage 20th anniversary special edition. NeuroImage. 2012;61(2): 386–396. 10.1016/j.neuroimage.2011.11.074 22166794

[93] Aru J, Suzuki M, Rutiku R, Larkum ME, Bachmann T. Coupling the state and contents of consciousness. Frontiers in Systems Neuroscience. 2019;13: 43 10.3389/fnsys.2019.00043 31543762 PMC6729974

[94] Sadaghiani S, Scheeringa R, Lehongre K, Morillon B, Giraud AL, et al Intrinsic connectivity networks, alpha oscillations, and tonic alertness: A simultaneous electroencephalography/functional magnetic resonance imaging study. J Neurosci. 2010;30(30): 10243–10250. 10.1523/JNEUROSCI.1004-10.2010 20668207 PMC6633365

[95] Dosenbach NU, Fair DA, Miezin FM, Cohen AL, Wenger KK, et al Distinct brain networks for adaptive and stable task control in humans. Proc Natl Acad Sci U S A. 2007;104(26): 11073–11078. 10.1073/pnas.0704320104 17576922 PMC1904171

[96] Yeo BT, Krienen FM, Sepulcre J, Sabuncu MR, Lashkari D, et al The organization of the human cerebral cortex estimated by intrinsic functional connectivity. J Neurophysiol. 2011;106(3): 1125–1165. 10.1152/jn.00338.2011 21653723 PMC3174820

[97] Golland Y, Bentin S, Gelbard H, Benjamini Y, Heller R, et al Extrinsic and intrinsic systems in the posterior cortex of the human brain revealed during natural sensory stimulation. Cereb Cortex. 2007;17(4): 766–777. 10.1093/cercor/bhk030 16699080

[98] Hyafil A, Fontolan L, Kabdebon C, Gutkin B, Giraud AL. Speech encoding by coupled cortical theta and gamma oscillations. Elife. 2015;4: e06213 10.7554/eLife.06213 26023831 PMC4480273

[99] Schoffelen JM, Gross J. Source connectivity analysis with MEG and EEG. Hum Brain Mapp. 2009;30(6): 1857–1865. 10.1002/hbm.20745 19235884 PMC6870611

[100] Cardinale F, Cossu M, Castana L, Casaceli G, Schiariti MP, et al Stereoelectroencephalography: Surgical methodology, safety, and stereotactic application accuracy in 500 procedures. Neurosurgery. 2013;72(3): 353–66. 10.1227/NEU.0b013e31827d1161 23168681

[101] Narizzano M, Arnulfo G, Ricci S, Toselli B, Tisdall M, et al SEEG assistant: A 3DSlicer extension to support epilepsy surgery.—BMC Bioinformatics. 2017;18(1): 124 10.1186/s12859-017-1545-8 28231759 PMC5324222

[102] Arnulfo G, Schenone A, Massimini M, Pigorini A, Nobili L, et al. A novel closest white-matter-contact-based referencing scheme for stereotactical EEG recordings. Front. Neuroinform. Conference Abstract: 5th INCF Congress of Neuroinformatics. 2014. 10.3389/conf.fninf.2014.08.00005

[103] Rouhinen S, Panula J, Palva JM, Palva S. Load dependence of beta and gamma oscillations predicts individual capacity of visual attention. J Neurosci. 2013;33(48): 19023–33. 10.1523/JNEUROSCI.1666-13.2013 24285906 PMC6618707

[104] Dale AM, Liu AK, Fischl BR, Buckner RL, Belliveau JW, et al Dynamic statistical parametric mapping: Combining fMRI and MEG for high-resolution imaging of cortical activity. Neuron. 2000;26(1): 55–67. 10.1016/s0896-6273(00)81138-1 10798392

[105] Gramfort A, Luessi M, Larson E, Engemann DA, Strohmeier D, et al MNE software for processing MEG and EEG data. Neuroimage. 2014;86: 446–460. 10.1016/j.neuroimage.2013.10.027 24161808 PMC3930851

[106] Taulu S, Simola J. Spatiotemporal signal space separation method for rejecting nearby interference in MEG measurements. Phys Med Biol. 2006;51(7): 1759–1768. 10.1088/0031-9155/51/7/008 16552102

[107] Korhonen O, Palva S, Palva JM. Sparse weightings for collapsing inverse solutions to cortical parcellations optimize M/EEG source reconstruction accuracy. J Neurosci Methods. 2014;226C: 147–160. 10.1016/j.jneumeth.2014.01.031 24509129

[108] Hirvonen J, Monto S, Wang SH, Palva JM, Palva S. Dynamic large-scale network synchronization from perception to action. Network Neuroscience. 2018;2(4): 442–463. 10.1162/netn_a_00039 30320293 PMC6175692

[109] Hillebrand A, Barnes GR. A quantitative assessment of the sensitivity of whole-head MEG to activity in the adult human cortex. Neuroimage. 2002;16(3): 638–650. 10.1006/nimg.2002.1102 12169249

[110] Wang SH, Lobier L, Siebenhühner F, Puoliväli T, Palva S, et al Hyperedge bundling: A practical solution to spurious interactions in MEG/EEG connectivity analyses. NeuroImage. 2018;S1053–8119(18): 0056. 10.1016/j.neuroimage.2018.01.056 29378318

[111] Bruns A. Fourier-, Hilbert- and wavelet-based signal analysis: Are they really different approaches? J Neurosci Methods. 2004;137(2): 321–332. 10.1016/j.jneumeth.2004.03.002 15262077

[112] Wechsler D. Wechsler adult intelligence scale–third edition. San Antonio, USA: The Psychological Corporation; 1997.

[113] Reitan RM. Validity of the trail making test as an indicator of organic brain damage. Percept Mot Skills. 1958;8(3): 271–276. 10.2466/pms.1958.8.3.271

[114] Emslie H, Wilson C, Burden V, Nimmo-Smith I, & Wilson BA, editors. Behavioural assessment of the dysexecutive syndrome for children. Bury St. Edmunds, UK: Thames Valley Test Company; 2003.

